# Efficacy of different protocols of non‐surgical periodontal therapy in patients with type 2 diabetes: A systematic review and meta‐analysis

**DOI:** 10.1111/jre.13327

**Published:** 2024-09-29

**Authors:** Stefano Corbella, Alice Alberti, Nikolaos Donos, Benedetta Morandi, Pinar Ercal, Luca Francetti, Elena Calciolari

**Affiliations:** ^1^ Department of Biomedical, Surgical and Dental Sciences Università degli Studi di Milano Milan Italy; ^2^ IRCCS Ospedale Galeazzi Sant'Ambrogio Milan Italy; ^3^ Centre for Oral Clinical Research Institute of Dentistry, Faculty of Medicine and Dentistry Queen Mary University of London London UK; ^4^ Department of Medicine and Surgery, Centre of Dentistry University of Parma Parma Italy

**Keywords:** glycated hemoglobin, periodontal diseases, periodontitis, systematic review, type 2 diabetes mellitus

## Abstract

The aim of the present systematic review of the literature and meta‐analysis was to evaluate the efficacy of different protocols of NSPT without any adjunctive therapy in subjects with type 2 diabetes, by considering clinical and patient‐centered outcomes. For the purposes of the study randomized controlled clinical trials with more than 3‐month follow‐up were searched in MEDLINE, EMBASE, and Cochrane Central. Then the articles were screened for inclusion and considered based on the protocols adopted, the outcome measure, follow‐up, and the level of glycemic control. A total of 23 articles about 22 studies were included. NSPT was more effective than just oral hygiene measures/no treatment in reducing periodontal probing depth (PPD) and clinical attachment loss (CAL) at 3 months (0.47 mm [0.29–0.65 mm] and 0.50 mm [0.24–0.76 mm], respectively) and 6 months (0.56 mm [0.28–0.84 mm] and 0.45 mm [0.13–0.77 mm], respectively for PPD and CAL) follow‐up (very low and low level of evidence). The meta‐analysis found no evidence of a difference between full‐mouth disinfection versus quadrant protocol clinical outcomes (very low level of evidence). One study found no evidence of a difference in periodontal clinical response between good versus poor glycemic control. Based on the results of the present research NSPT protocols could be considered more efficacious than others in terms of clinical outcomes in subjects with type 2 diabetes. Moreover, NSPT resulted in efficacious improvement of periodontal parameters and HbA1c levels compared to no treatment or oral hygiene instructions alone.

## Graphical abstract

The study focused on the efficacy of different NSPT protocols in subjects with type 2 diabetes and periodontitis.NSPT was more efficacious than oral hygiene alone or associated with supra‐gingival scaling with regard to all periodontal clinical outcomes, including a small effect on HbA1c.

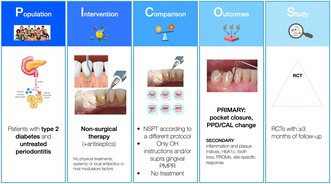

## 1 INTRODUCTION

Diabetes is one of the most prevalent non‐communicable diseases, being a chronic and metabolic condition characterized by elevated levels of blood glucose which leads, over time, to an increase in the risk of having health‐related complications and disorders such as retinopathy,^1^ cardiovascular disease,^2^ neuropathy,^3^ and nephropathy.^4^

Despite more than 101 years having passed since the first injection of insulin was successfully administered for controlling diabetes^5^ and many treatments are now available to manage this condition, in 2019 diabetes was the ninth cause of death in the world, being the direct or indirect cause of approximately 1.5 million deaths.^6^ According to the International Diabetes Federation, one in ten people in the world is affected by diabetes. Both the number of cases and the prevalence of diabetes have been steadily increasing over the past few decades.^5^

The bidirectional relationship between diabetes and periodontitis is well‐known and proved by robust scientific literature,^7^, ^8^ so it has been suggested that diabetes‐associated periodontitis should not be regarded as a distinct diagnosis, but diabetes should be recognized as an important modifying factor and included in the clinical diagnosis of periodontitis as a descriptor.^9^ On one side, diabetes is one of the most important risk factors for periodontal diseases, increasing by three to four times the risk of developing periodontitis as compared to healthy subjects.^8^, ^10^, ^11^ Moreover, it is known that systemic hyperglycemia is a significant independent risk factor for tooth loss.^12^, ^13^ A longitudinal study on 2198 subjects found a significant, direct correlation between glycemic control and mean periodontal probing depth (PPD) and clinical attachment loss (CAL).^14^ The bidirectional correlation between diabetes and periodontitis has its own basis in the common linkage with inflammatory mechanisms and the role played by oxidative stress.^15^, ^16^, ^17^, ^18^ The existence of a difference in oral microbiome between diabetic and non‐diabetic subjects is still controversial.^19^, ^20^, ^21^ Moreover, hyperglycemia impairs periodontal healing by decreasing the immune response and increasing oxidative stress.^15^, ^16^

The treatment protocol of subjects with periodontitis requires a stepwise approach, and one of the steps of therapy is represented by nonsurgical treatment with the aim of controlling subgingival biofilm and calculus.^17^ Recent systematic reviews of the literature highlighted that subgingival instrumentation is effective in reducing signs and symptoms of periodontal inflammation, leading to a mean PPD reduction of 1.4 mm after 6/8 months, and pocket closure ranging from 63.9% to 74%.^18^, ^19^ Numerous studies have previously explored the efficacy of periodontal treatment in improving diabetes‐related parameters, focusing mainly on glycemic control and insulin resistance.^20^, ^21^, ^22^ The recently updated Cochrane review by Simpson et al.^20^ analyzed 35 studies with 3249 participants, finding moderate‐certainty evidence that periodontal treatment can lower hemoglobin A1c (HbA1c) levels by 0.50% at 12 months. However, we have very limited evidence on how different NSPT protocols work in subjects with diabetes with reference to clinical outcomes and even the most recent systematic review on the topic did not provide details about different outcomes and comparability among different protocols.^23^ Considering the link between periodontitis and diabetes, it becomes of outmost importance to have a robust and evidence‐based understanding of how to manage diabetic patients affected by periodontitis to ensure predictable outcomes. Therefore, the aim of the present systematic review of the literature was to evaluate the efficacy of different protocols of NSPT without the use of adjunctive therapies such as physical treatments, systemic or local antibiotics, or host modulator factors in periodontitis patients with type 2 diabetes mellitus in terms of periodontal clinical outcomes.

## 2 METHODS

The protocol of the study was registered in the PROSPERO database with the number [CRD42021237742] before the initiation of the study. The protocol followed the instructions provided by the Cochrane Handbook for Systematic Review of Interventions—Second Edition^24^ and the results will be presented following the Preferred Reporting Items for Systematic Reviews and Meta‐Analysis (PRISMA) guidelines.^25^

The aim of this review is to answer the following focused question: in periodontitis patients with type 2 diabetes mellitus what is the efficacy of different protocols of non‐surgical periodontal therapy without the use of adjunctive therapies such as physical treatments, systemic or local antibiotics, or host modulators factors in terms of pocket closure, PPD reduction, CAL gain, and secondary outcomes such as gingival inflammation indexes, plaque indexes, number of teeth lost, and patient‐reported outcomes.

### 2.1 Eligibility criteria

The criteria for considering studies for this review based on the PICOS were as follows:

1. Population (P): ≥18 years old, untreated periodontitis patients (defined following the current and past classifications^26^, ^27^, ^28^, ^29^ as Stage II, Stage III, or Stage IV periodontitis (any grade) or mild, moderate, or severe periodontitis) affected by controlled or uncontrolled type 2 diabetes (code 5A11 following the International Classification of Diseases of the World Health Organization^30^).
2. Intervention (I): Sub‐gingival instrumentation not associated with therapies such as physical treatments, systemic or local antibiotics, or host modulator factors. The use of local antiseptics was considered.
3. Control (C): (a) The same sub‐gingival instrumentation performed according to a different protocol in patients with controlled or uncontrolled diabetes (e.g., different number of visits or different types of instruments); (b) oral hygiene instructions alone or with supragingival scaling; and (c) no treatment.
4. Outcomes (O):
  - Primary outcomes:
    - Percentage or number of pockets closed (defined as PPD < 5 mm and no PPD = 4 mm with bleeding on probing (BOP)); reduction in PPD, which is defined as the distance from the gingival margin to the base of the pocket; changes in CAL, which is the measurement of the position of the soft tissue in relation to cemento‐enamel junction.
  - Secondary outcomes:
    - Site‐specific response to subgingival instrumentation (in horizontal defects, intrabony defects, and furcations).
    - Changes in gingival inflammation indexes (e.g., Gingival Bleeding index, Gingival index (GI), and percentage of bleeding sites (BOP)).
    - Changes in plaque indexes (e.g., Plaque index (PI), Turesky‐modified plaque index, and proportion of sites with visible plaque).
    - Changes in HbA1c concentrations.
    - Number of teeth lost or extracted during the examination period.
    - Patient‐reported outcome measures (PROMs), including adverse events.
5. **Studies (S):** Randomized controlled clinical trials with at least 3‐month follow‐up. Split‐mouth studies were excluded.

### 2.2 Search and study selection

The electronic search for pertinent articles was performed by interrogating the following electronic databases: MEDLINE/PubMed, EMBASE, and Cochrane Central using a search strategy presented in Appendix S1. Grey literature was searched interrogating Greylit and OpenGrey. Trials registers (ClinicalTrials.gov and EU Clinical Trials Register) were also searched. The manual search of all potentially relevant papers was performed from the reference lists of included papers and of all the issues published since 1990 of the journals in the following list: Journal of Clinical Periodontology, Journal of Periodontology, Journal of Periodontal Research, Journal of Dentistry, and Journal of Dental Research. No language limitations were posed. Conference papers and abstracts were excluded. Scopus was consulted checking the articles citing the papers included.

The last electronic search was performed on July 17, 2023.

Two reviewers (SC and EC) independently screened titles and abstracts for a preliminary check of all the inclusion criteria. The second stage of articles selection was performed by the same authors, by carefully screening the full texts of the identified papers. In case of disagreement a third reviewer (ND) was interrogated to solve the dispute. Reasons for exclusion in the second step were recorded, and the level of concordance in each step of the selection process was assessed through Cohen's kappa.

The unit of interest was the study, rather than the report, thus multiple reports of the same studies were identified and linked after data extraction as suggested by the Cochrane Handbook.^31^

### 2.3 Data extraction

The process of data extraction was performed independently by two authors (AA and PE) to retrieve the following information, when available: authors' names, year of publication, country, characteristics of the sample (age distribution, sex distribution, ethnicity, educational status, and smoking status), characteristics of the diabetes (definition and type, level of control of the disease, HbA1c levels changes, and drugs), definition/assessment of periodontitis, characteristics of the periodontal treatment, clinical data before and after the treatment, including number of teeth lost, percentage or number of pockets closed, mean PPD, mean CAL, gingival bleeding indexes (e.g., Gingival Bleeding index, Gingival index (GI), and percentage of bleeding sites (BOP)), plaque indexes (e.g., PI, Turesky‐modified plaque index, and proportion of sites with visible plaque) or difference between baseline and follow‐up values, occurrence of adverse events or complications, and PROMs.

In case of missing information, the authors were contacted by email up to twice for providing missing data.

### 2.4 Risk of bias evaluation and quality of evidence assessment

The risk of bias evaluation and the quality of evidence assessment were performed independently by two reviewers (SC and LF) and any disagreement resolved by discussion.

The criteria for evaluating the risk of bias in included studies were the ones of the Cochrane risk‐of‐bias tool for randomized trials 2.0^24^:

1. Bias arising from the randomization process.
2. Bias due to deviations from intended interventions.
3. Bias due to missing outcome data.
4. Bias in the measurement of the outcome.
5. Bias in the selection of the reported result.

The overall risk‐of‐bias judgment was considered as *high risk* if the level of risk of bias is high for at least one domain or if the trial is judged to have some concerns for multiple domains (three). If the trial was judged to have some concerns for less than three domains the overall risk of bias was “some concerns,” while the study had a *low risk* of bias if all domains were judged to have low risk.

The funding bias was estimated by evaluating if authors disclosed their potential sources of competing conflict of interest and the source of funding for the studies they carried on (if any).

Moreover, the quality of the available evidence was assessed for each comparison and for each outcome in the meta‐analysis using the Grading of Recommendations, Assessment, Development, and Evaluations (GRADE) approach.^32^

### 2.5 Summary measures and synthesis of the results

To perform a meta‐analysis, studies were grouped on the basis of the treatments that were carried out, follow‐up time, and, whenever possible, on the basis of the type and the level of control of diabetes. The meta‐analysis was performed using the software RevMan (Review Manager Version 5.3, 2014; The Nordic Cochrane Center, The Cochrane Collaboration, Copenhagen, Denmark) if at least three studies were available for each comparison. Subgroup analysis in the context of the same comparison was performed to account for different characteristics of the control treatment performed, namely by grouping studies with a null control group (no treatment), with only administration of oral hygiene instructions (OHI), and with administration of OHI and supragingival scaling.

For each continuous outcome, the difference between baseline and follow‐up values was extracted with its specific error measure (standard deviation, standard error, or variance). When difference values were not reported, they were calculated as the difference between baseline and follow‐up values, and error (namely standard deviation) was computed following the procedure described in Appendix S2. In the meta‐analysis effect size was computed through the weighted mean method and results were combined using the DerSimonian and Laird's random‐effect model,^33^ assuming heterogeneity among studies.

Cochran's test serves to measure the consistency of the results, considering it significant if *p* < .01. *I* ^2^ statistics was applied to measure heterogeneity (total variation across studies that was due to heterogeneity rather than to chance). If *I* ^2^ is less than 40% the heterogeneity is negligible, if it is from 40% to 60% the heterogeneity is moderate, if it is from 60% to 90% there is substantial heterogeneity, while if it is from 75% to 100% there is a considerable heterogeneity.^24^

Small study effects, as a proxy for publication bias, were assessed by testing for funnel plot asymmetry and by calculating Egger's bias, as described in the Cochrane Handbook.^24^

A sensitivity analysis was performed by excluding from the meta‐analysis studies that were at high risk of bias and considering only studies not using any type of local antiseptics, such as chlorhexidine.

## 3 RESULTS

**FIGURE 1:**
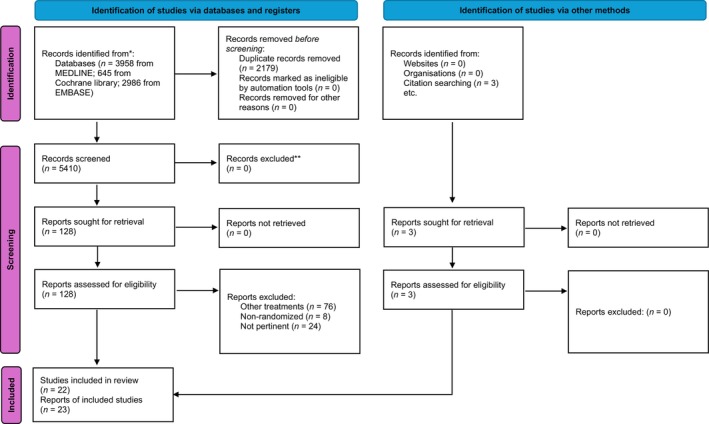
PRISMA diagram of the article selection process. *Consider, if feasible to do so, reporting the number of records identified from each database or register searched (rather than the total number across all databases/registers). **If automation tools were used, indicate how many records were excluded by a human and how many were excluded by automation tools. From: Page et al., 2021.^71^

The article selection process is summarized in Figure 1. A total of 23 papers, accounting for 22 studies, were included in the qualitative synthesis.^34^, ^35^, ^36^, ^37^, ^38^, ^39^, ^40^, ^41^, ^42^, ^43^, ^44^, ^45^, ^46^, ^47^, ^48^, ^49^, ^50^, ^51^, ^52^, ^53^, ^54^, ^55^, ^56^

Seven studies were performed in Brazil,^35^, ^38^, ^39^, ^41^, ^42^, ^47^, ^48^ four studies in China,^36^, ^44^, ^50^, ^53^ three in India,^34^, ^43^, ^49^ one in the United States,^40^, ^45^ Japan,^51^ Malaysia,^46^ Taiwan,^54^ Vietnam,^55^ Italy,^56^ Spain,^52^ and Iran.^37^ A total of 1548 patients were assessed, with a follow‐up time ranging from 3 months to 2 years. The included studies considered heterogeneous definitions of periodontitis: the majority of them considered subjects with moderate or severe periodontitis,^34^, ^40^, ^45^, ^46^, ^47^, ^48^, ^51^, ^54^, ^55^ mild or moderate periodontitis,^37^, ^43^, ^49^ one study specified that stages III and IV periodontal patients were included,^53^ while some papers reported a generic diagnosis of chronic periodontitis.^35^, ^36^, ^38^, ^39^, ^41^, ^42^, ^44^, ^50^, ^52^, ^56^ In all the samples, no changes in the diabetes medication regimen were adopted or suggested. In four studies chlorhexidine was used as a local antiseptic.^42^, ^43^, ^46^, ^55^ A summary of the characteristics of the studies is presented in Table 1.

**TABLE 1:** Characteristics of the included studies.

Authors, year of publication, country of the study	Study type	Number of subjects; sex (M/F)	AGE (mean ± SD (range))	Systemic conditions/Health status	Ethnicity	Periodontitis	Diabetes	Outcomes	Follow UP	Type of probe and N sites/tooth evaluated	Group 1	Group 2	Group 3	Group 4	Group 5
Chen et al. 2012 China	Parallel RCT	134; 68/66 G1: 23/19 G2: 26/17 G3: 17/24	G1: 59.86 ± 9.48 G2: 57.91 ± 11.35 G3: 63.2 ± 8.51	Excluded: other systemic disease influencing periodontal disease, ATB in the previous 3 mo, active infections, pregnancy/lactation	NR	CP (AAP) with a ≥1 mm mean CAL loss	T2D for >1 year, no changes in diabetes therapy in the previous 2 mo, no major diabetic complication	Change in PPD, REC, CAL, BOP, PI, % of sites with PPD ≤ 3 mm, PPD = 4–5 mm, and PPD ≥6 mm, HbA1c, hsCRP, FPG, TC, TG, HDL‐C, LDL‐C, TNF‐a	1.5, 3, 6 mo	6 sites per tooth	SRP + additional subgingival debridement at 3 mo follow‐up; Standard Gracey curettes + ultrasonic instrument	SRP + supragingival prophylaxis in deep periodontal pockets at 3 mo	No treatment	—	—
Engebretson et al. 2013 USA	Multi‐center RCT, single blind	514; 277/237 G1: 143/114 G2: 134/123	G1: 56.7 ± 10.5 G2: 57.9 ± 9.6	Excluded: nonsteroidal anti‐inflammatory drugs other than daily low‐dose aspirin (75–325 mg), immunosuppressive drugs, pregnancy	Black 146, White 280, Hispanic 166, other 88	Moderate/severe CP, ≥5 mm in 2 or more quadrants of the mouth	T2D for ≥3 mo, no changes in diabetic medications in the previous 3 mo HbA1c 7.0%–9.0%	Change in PPD, CAL, BOP, PI, GI, HbA1c, FPG, fasting insulin level, HOMA2 insulin resistance, HOMA2 β‐cell function, weight, blood pressure	3 and 6 mo	PPD, CAL, and BOP: 6 sites per tooth, PI and GI at 6 indexed teeth	SRP at baseline, 3 and 6mo; hand and sonic or ultrasonic instruments	OHI	—	—	—
Hsu et al. 2021	RCT, double‐blinded	68 (35 test: 18 M/17 F; 33 control 22 M/11F)	Test: 54,7 ± 6,1 Control: 54.8 ± 6.9	Excluded: periodontal treatment in last 6 mo, systemic diseases, routine AB use or bisphosphonates	NR	Severe periodontal status with at least one PPD ≥5 mm, BoP, at least 16 teeth in the mouth	T2D	Change in PPD, CAL, GI, BOP, PI, OHQoL, HbA1c	1, 3, 6 mo	CAL, BOP, PPD 6 sites/tooth; GI and PI 4 sites around 6 teeth	NSPT	NSPT + Community Health Worker (CHW) strategy on oral self‐care behavior (30 min‐ in each NSPT session lessons on OHI instructions and period care related to diabetes)	—	—	—
Kaur et al. 2015 India	RCT, single‐blinded (examiner)	100; G1: 10/13 G2: 12/15 G3: 14/11 G4: 12/13 G5: 15/10	G1: 52.83 ± 5.04 G2: 50.96 ± 6.43 G3: 54.28 ± 5.93 G4: 51.60 ± 5.93 G5: 51.56 ± 5.91	Excluded: cardiovascular disease, chronic respiratory disease, rheumatoid arthritis, or any other systemic disease that could influence the course of periodontal disease, current/former smokers, no ATB in the previous 3 mo, pregnancy/lactation	NR (no differences between groups)	Moderate CP (≥2 interproximal sites in different teeth with CAL loss ≥4 mm or PPD ≥5 mm), severe CP (≥2 interproximal sites in different teeth with CAL loss ≥6 mm, or ≥1 interproximal sites in different teeth with PPD ≥5 mm) (Page & Eke 2007) (Page RC, Eke PI (2007) Case definitions for use in popu‐ lation‐based surveillance of periodontitis. J Periodontol 78, 1387–1399); ≥12 remaining teeth (excluding third molars), no periodontal treatment during the previous 6 mo	T2D for ≥1 year, no major diabetic complications, no change in medication use in the previous 2 mo or during the study	Change in PPD, CAL, REC, BOP, PI, GI, HbA1c, blood glucose	3 and 6 mo	Williams Probe 6 sites per tooth except third molars	G1 (good glycemic control with HbA1c < 7%): SRP in 4 sessions within 2 weeks + OHI; Standard Gracey curettes + ultrasonic instrument	G2 (poor glycemic control with HbA1c ≥ 7%): SRP in 4 sessions within 2 weeks + OHI; Standard Gracey curettes + ultrasonic instrument	G3 (good glycemic control with HbA1c < 7%): no treatment	G4 (poor glycemic control with HbA1c ≥ 7%): no treatment	G5 (non‐diabetic patients): SRP in 4 sessions within 2 weeks + OHI *Not considered in the present review
Michalowicz et al. 2014 USA [Engebretson et al. 2013]	Multi‐center RCT, single‐blind	514; 277/237 G1 143/114 G2 134/123	G1 56.7 ± 10.5 G2 57.9 ± 9.6	Excluded: pregnancy, use of nonsteroidal anti‐ inflammatory drugs other than daily low‐dose aspirin (75–325 mg), use of immunosuppressive medications	Black 146, White 280, Hispanic 166, other 88	Moderate or severe CP, ≥5 mm in 2 or more quadrants of the mouth	T2D for ≥3 mo, no changes in diabetic medications in the previous 3mo HbA1c 7.0%–9.0%	Change in PPD, CAL, BOP, PI, GI, HbA1c, FPG, fasting insulin level, HOMA2 insulin resistance, HOMA2 β‐cell function, weight, blood pressure	3 and 6 mo	PPD, CAL, and BOP: 6 sites per tooth, PI and GI at 6 indexed teeth	SRP at baseline, 3 and 6 mo; powered scalers and hand curettes	OHI	—	—	—
Mizuno et al. 2017 Japan	Parallel RCT, single‐blind (examiner); randomization was stratified by levels of HbA1c, use of insulin and number of medications	40; 28/9 Test group: 13/7 Control group: 15/2	Test group: 61.2 ± 9.2 Control group: 62.8 ± 12.1	Excluded: limited life expectancy, pregnancy	NR	Moderate or severe CP (≥2 interproximal sites with CAL loss ≥3 mm and ≥2 interproximal sites with PPD≥4mmm on different teeth or ≥1 site with PPD≥5 mm), no periodontal treatment in the previous 6 mo	T2D ≥ 2mo, no changes in diabetes therapy during the trial unless medically indicated, no major diabetes complication	Change in PPD, CAL, BOP, Plaque Control Record, HbA1c, serum levels of glycated albumin, lipids and creatinine, serum ROMs	3 and 6 mo	CP‐11 Color‐Coded Probe 6 sites per tooth	Supragingival plaque removal + OHI	SRP in multiple sessions within 42 days; Curettes + ultrasonic instrument	—	—	—
Moeintaghavi et al. 2012 Iran [just info about control]	RCT, single‐blind (examiner)	40; 22 test, 18 control	Given as overall: 50.29 ± 3	Excluded: presence of systemic diseases other than DM2 that could influence the course of periodontal disease; intake of immunosuppressive drugs, steroids, hydantoin or non‐steroidal anti‐inflammatory drugs; tobacco use; pregnancy or intention to become pregnant during the study period; fixed orthodontic appliances	NR	Mild to moderate periodontitis according to AAP	Diagnosis of DM2 with glycated hemoglobin (HbA1c) values over 7%; no major diabetic complications; blood sugar controlled with glyben glamide and metformin, without insulin administration	Change in PPD, CAL, PI, GI, HbA1c, fasting plasma glucose, high‐density lipoprotein cholesterol, LDL, TG, TC	3 mo	Williams probe; 4 sites per tooth for PI, sites for other indices not given	Full mouth scaling and root planing with both ultrasonic and hand instruments (test)	No treatment	—	—	—
Nassar et al. 2012 Brazil	RCT	20; NR	18–70	Patients with lipid alterations. Excluded: smoking in the previous 5 years, ATB and hyperlipidemia control medication in the previous 6 mo, anti‐inflammatory drugs in the last 3 mo, hormone therapy, pregnancy	NR	CP, ≥ 2 sites with PPD >4 mm, CAL ≥3 mm, BOP+ and gingival inflammation	T2D	Change in PPD, CAL, PI, GI, TC, Cholesterol fractions, TG	6 mo, 2 years	Williams type No. 23 6 sites per tooth	SRP	SRP + maintenance therapy	—	—	—
Nassar et al. 2014 Brazil	RCT	40; NR	NR	Excluded: smoking in the last 5 years, ATB in the previous 6 mo, anti‐inflammatory in the last 3 mo, hormone therapy, pregnancy	NR	CP, ≥4 sites with PPD≥5 mm, BOP+ and gingival inflammation	T2D Good metabolic control HbA1c > 6.5 mg/dL	Change in PPD, CAL, BOP, Hb1Ac	3 and 6 mo	Williams type No. 23 6 sites per tooth	SRP in multiple sessions	FMD	—	—	—
Raman et al. 2014 Malaysia	RCT	32; 15 (test), 17 (control); 11/4, 9/8 (M/F)	Test group: 57.7 ± 9.9 Control group: 54.6 ± 6.2	Excluded: a history of systemic ATB usage over the previous 4 mo, having received non‐surgical periodontal treatment within the past 6 mo or surgical periodontal treatment within the past 12 mo; pregnancy, change of medication for diabetes during the course of the study, current smokers or history of a cerebrovascular or cardiovascular event within the past 12 mo	Malay (33% T, 23.5% C); Chinese (26.7% T, 23.5% C); Indian (40% T, 52.9% C)	Moderate to advanced chronic periodontitis with at least 12 teeth present and with 5 or more pockets of 5 mm or more and probing attachment loss of 4 mm or more in at least 2 different quadrants which bled on probing	T2D diagnosed at least 1 year prior to study	Change in PPD, PI, GBI, Probing attachment loss (CAL), HbA1c, hs‐CRP	2 mo, 3 mo	Florida Probe/NR	Full mouth debridement (SRP in a single visit) using an ultrasonic device and Gracey curettes, CHX for 14 days	OHI	—	—	—
Santos et al. 2009 Brazil	RCT	36; 16/20 G1: 8/10 G2: 8/10	G1: 52.3 G2: 53	Excluded: smoking, other systemic diseases affecting periodontal status, ATB in the previous 6 mo, long‐term immunosuppressive drugs, anti‐inflammatory drugs, pregnancy/lactation	NR	CP (1999 classification), >30% sites with PPD and CAL ≥5 mm	T2D for ≥5 years	Change in PPD, CAL, BOP, PI, suppuration, HbA1c, FPG	3 and 6 mo	6 sites per tooth	SRP in 2 sessions within 24 h	SRP in 4 sessions within 21 days	—	—	—
Santos et al. 2012 Brazil [just info about control]	Parallel RCT, single‐blind	34; 16/18 G1: 9/8 G2: 7/10	G1: 51.9 ± 7.8 (42–66) G2: 53.1 ± 8.1 (42–67)	Excluded: other systemic conditions that could affect the progression of periodontal disease, smoking in the previous 5 years, ATB in the previous 6 mo, regular use of mouth rinses containing antimicrobials in the previous 2 mo, long‐term treatment with anti‐inflammatory and immunosuppressive medications, pregnancy/lactation	NR	CP, ≥30% of sites with a PPD and CAL ≥4 mm	T2D for ≥5 years, no multiple systemic diabetic complications	Change in PPD, CAL, BOP, PI, suppuration, % of sites with PPD ≥5 mm, FPG, HbA1c), levels of cytokines, and osteoclastogenesis‐related factors	3, 6, 12 mo	UNC probe 6 sites per tooth	SRP in 2 sessions within 24 h + maintenance therapy at 3, 6, and 9 mo	SRP in 4 sessions within max 21 days + maintenance therapy at 3, 6, 9 mo	—	—	—
Santos et al. 2013 Brazil	Parallel RCT, single‐blind	34; 10/28 G1: 4/15 G2: 6/13	G1: 37–70; 50.3 ± 9.5 G2: 35–75; 53.9 ± 10.8	Excluded: pregnancy/lactation, smoking in the previous 5 years, ATB in the previous 6 mo, use of mouth rinses containing antimicrobials in the previous 3 mo, other systemic conditions affecting periodontitis, long‐term administration of anti‐inflammatory and immunosuppressive medications	NR	Generalized CP (Armitage 1999), >30% of the sites with concomitant PPD and CAL >4 mm	T2D for ≥5 years, no major diabetic complication	Change in PPD, CAL, BOP, PI, suppuration, HbA1c, FPG	3, 6, 12 mo	UNC probe 6 sites per tooth excluding third molars	FMD within 24 h + local application of CHX gel + CHX rinses for 60 days	SRP within 24 h + local application of placebo gel + placebo rinses for 60 days	—	—	—
Singh et al. 2008 India	RCT	45, NR	NR	Exclusion: uncontrolled DM, periodontal treatment in last 6 mo, ATB history in the last 3 mo, less than 16 remaining teeth	NR	Moderate or severe periodontitis, 30% or more of the teeth examined having ≥4 mm probing depth	T2D	Change in PPD, CAL, GI, PI, HbA1c, fasting plasma glucose levels, 2‐h postprandial glucose	3 mo	NR	No treatment	Full mouth SRP + OHI	Full mouth SRP + OHI + systemic doxycycline	—	—
Telgi et al. 2013 India	RCT	60, NR	35–45 years	Exclusion: no systemic diseases other than diabetes, no ATB use and perio treatment in last 6 mo, tobacco and alcohol use	NR	Mild to moderate periodontitis, PPD of 4–5 mm, presence of a minimum of 28 teeth	T2D, with oral hypoglycemic agents	Change in PPD, GI, PI, HbA1c, FBS	3 mo	UNC‐15 probe/6 points	Scaling +0.12% CHX once daily + brushing	0.12% CHX once daily + brushing	Brushing only	—	—
Toyama et al. 2014 Brazil	RCT	20; NR 43.2/56.8%	44.5 ± 2.5	Excluded: smoking, ATB in the previous 6 mo, anti‐inflammatory drugs in the previous 3 mo, pregnancy	NR	Moderate or severe CP, ≥ 6 sites with PPD >5 mm, CAL ≥4 mm, BOP+, ≥20 remaining teeth	T2D HbA1c >7%	Change in PPD, CAL, BOP, Hb1Ac, FPG, and GCF levels of IL‐β	3 and 6 mo	Williams‐type periodontal probe No. 23 6 sites per tooth	SRP by quadrant/sextant in multiple sessions	FMD	—	—	—
Wang et al. 2020 China	RCT, single‐blind	58;33/25 17/12; 16/13	64.4/64.7	Excluded: ATB, anti‐inflammatory agents or immunosuppressants in the previous 3 mo, requirement of ATB prophylaxis, pregnancy, no periodontal treatment in the previous 6 mo	NR	Stage III/IV periodontitis (≥6 sites with PPD ≥4 mm; > 25% of interproximal sites with CAL loss ≥5 mm), ≥8 remaining teeth	T2D ≥ 5 years (WHO/IDF 2006), no changes in diabetes therapy in the previous 6 mo HbA1c > 6.5%	Change in PPD, BOP, CAL, PI, HbA1c, CRP, IL‐6, NT‐proBNP, LVMI, echocardiography (E/e ratio)	6 mo	UNC‐15 6 sites per tooth	SRP; hand instruments + piezoelectric ultrasonic scaler	OHI	—	—	—
Wu et al. 2015 China	RCT	46 (Test 23. Control 23); 12/11 T, 10/13 C M/F ratio	Test group: 54.09 ± 6.57 Control group: 55.52 ± 5.22	Excluded: no anti‐inflamatory medications, no perio treatment in last 6 mo, aggressive periodontitis, current or past tobacco users, other systemic diseases that can alter periodontal diseases	NR	CP, mean AL ≥1 mm according to AAP	T2D diagnosed over a year ago, no medication changes in the last 3 mo	Change in PPD, CAL, BOP, GI, HbA1c, visfatin	3 mo, 6 mo	NR	SRP + OHI	OHI only	—	—	—
Zhang et al. 2013 China	RCT, single‐blind (examiner)	71 (test 49, control 22) 21/27 T, 10/12 C M/F ratio	Test group: 60.4 ± 9.77 Control group: 62.7 ± 10.7	Excluded: accompanied with other systemic immune diseases; administered with ATB, immunomodulators, contraceptives, or any other form of hormone within the past 3 mo; underwent modified diabetes treatment strategy within 3 mo; had periodontal treatment within the past 12 mo; needed extraction or endodontic treatment; smokes more than four cigarettes per day; pregnant or lactating women	NR	CP, at least four teeth with probing pocket depth ≥5 mm, CAL ≥4 mm, and BOP, distributed in two or more oral quadrants	T2D: 1) postprandial plasma glucose ≥200 mg/dL (11.1 mmol/L); (2) fast plasma glucose (FPG) ≥126 mg/dL (7.0 mmol/L); (3) 2‐h oral glucose tolerance test ≥200 mg/dL (11.1 mmol/L). HbA1c at least 5.5% 3 mo before recruitment	Change in HbA1c, FPG level, PPD, BOP, PI, CAL	3 mo	Williams probe/6 sites per tooth	No treatment	SRP; supra−/subgingival ultrasonic scaling + manual curettage	—	—	—
Pham et al. 2022 Vietnam	RCT	42; G1: 21/20 G2: 21/20	54.7 ± 7.4; G1: 53.5 ± 8.0; G2: 55.1 ± 6.8	Excluded: not following the protocol, aggressive periodontitis or periodontitis associated with endodontic lesion, NSPT in the previous 6 mo, acute conditions or immunosuppressive medications, major diabetic complications	NR	At least 3 teeth diagnosed with moderate/severe periodontitis according to AAP: each tooth with one bleeding site, PPD ≥5 mm or CAL loss ≥3 mm and ABL ≥16% or >3 mm	T2D No major diabetes complication	Change in PPD, CAL, PI, BI, BoP%, FPG, HbA1c, CRP	1, 3, and 6 mo	UNC probe 6 sites per tooth	SRP FMD, with irrigation with PVP‐I 10% and 0.05% CHX rinses for 14 days; at 1, 3 and 6 mo supragingival scaling, at 3 and 6 mo rinses 0.05% CHX	Supragingival scaling and 0.05% CHX rinses for 14 days; at 3 and 6 mo rinses	—	—	—
Graziani et al. 2023 Italy	RCT	40; G1: 20/20, 12 male; G2: 20/19, 12 male	G1: 62.45 ± 9.60; G2: 56.89 ± 10.37	Excluded: pregnant or lactating, other systemic diseases, any pharmacological treatment within 3 mo except for diabetes medications, periodontal treatment in the last 6 mo	NR	Proximal attachment loss ≥3 mm in ≥2 non‐adjacent teeth, at least 20% of the entire dentition with PPD ≥5 mm	T2D HbA1c > = 48 mmol / mol	Change in PPD, CAL, REC, BoP%, FMPS%, CRP, IL‐6, cholesterol, triglycerides, HbA1c, insulin, glucose levels, vital signs (systolic and diastolic blood pressure, heart rate, body temperature), endothelial function, OHIP‐14	3 mo	UNC probe 6 sites per tooth	Ultrasonic SRP, quadrant in multiple sessions	Ultrasonic SRP, FMD	—	—	—
Mauri‐Obradors et al. 2018 Spain	RCT	90; G1: 48/44, 20 male; G2: 42/35, 17 male	G1: 62 ± 10; G2: 61 ± 11	Excluded: ATB treatment during the previous 15 days or for periods >10 days during the last 3 mo, non‐surgical treatment within the past 6 mo, pregnancy, changes in diabetes medication during the study, ASA III or IV	NR	Generalized CP, At least nine teeth present and >30% of the probed gingiva with PPD and CAL ≥4 mm (Armitage 1999)	T2D	Change in PPD, PI, GI, CAL, HbA1c, FPG	6 mo	NR	Supragingival ultrasonic scaling	Full‐mouth supragingival and subgingival debridement; Ultrasound + Gracey curettes	—	—	—
Artese et al. 2015 Brazil	RCT	24; G1: 12, 56.3% women; G2: 12, 52.0%	G1: 54.4 ± 5.8; G2: 52.0 ± 3.3	Excluded: pregnant women, smokers, BMI > 35 kg/m2, previous periodontal treatment, systemic ATB or antiseptic within 6 mo	NR	Generalized severe periodontitis, PPD in 30% sites, CAL >4 mm, and BOP	T2D diagnosed according to WHO	Change in Visible plaque index (VPI), GBI, BOP, PPD, CAL, HbA1c, IL‐6, IL‐8, IL‐17, TNF‐a, MCP‐1	6 mo	UNC probe	Supragingival ultrasonic scaling and curettes	Supragingival and subgingival scaling in two appointments	—	—	—

Abbreviations: AAP, American Academy of Periodontology; ATB, antibiotics; BOP, bleeding on probing; CAL, clinical attachment loss; CHW, community health worker; CHX, chlorhexidine; CP, chronic periodontitis; F, female; FMD, full mouth disinfection; FPG, fasting plasma glucose; GBI, Gingival Bleeding Index; GI, gingival index; HbA1c, glycated hemoglobin; HDL‐C, high‐density lipoprotein cholesterol; HOMA2, homeostatic model assessment 2; hsCRP, high‐sensitivity C‐reactive protein; hs‐CRP, high‐sensitivity C‐reactive protein; IL‐17, interleukin‐17; IL‐6, interleukin‐6; IL‐8, interleukin‐8; LDL‐C, low‐density lipoprotein cholesterol; LVMI, Left Ventricular Mass Index; M, male; MCP‐1, monocyte chemoattractant protein‐1; mo, month; NR, not reported; NSPT, non‐surgical periodontal therapy; NT‐proBNP, N‐terminal pro‐brain natriuretic peptide; OHI, Oral Hygiene Index; OHRQoL, Oral Health‐Related Quality of Life; PI, Plaque Index; PPD, pocket/periodontal probing depth; REC, recession; ROMs, reactive oxygen metabolites; RTC, randomized controlled trial; SD, standard deviation; SRP, scaling and root planing; T2D, type 2 diabetes; TC, total cholesterol; TG, triglycerides; TNF‐a, tumor necrosis factor‐alpha; UNC probe, University of North Carolina probe.

### 3.1 Risk of bias evaluation

The results of the risk of bias evaluation are reported in Table 2 and in Appendix S3. Two studies were considered to be at high risk of bias, one due to missing outcomes data,^51^ and one due to methods of allocation.^55^ Thirteen studies presented some concerns about risk of bias^34^, ^35^, ^36^, ^37^, ^38^, ^39^, ^41^, ^42^, ^43^, ^46^, ^47^, ^50^, ^54^ and seven were at low risk of bias.^40^, ^44^, ^48^, ^49^, ^52^, ^53^, ^56^

**TABLE 2:** Results of risk of bias evaluation.

Study	Randomization process	Deviations from intended interventions	Missing outcome data	Measurement of the outcome	Selection of the reported result	Overall bias	Notes
Artese et al. 2015	Low	Low	Low	Low	Low	Low	
Chen et al. 2012	Low	Some concerns	Low	Low	Low	Some concerns	
Engebretson et al. 2013	Low	Low	Low	Low	Low	Low	
Graziani et al. 2023	Low	Low	Low	Low	Low	Low	
Hsu et al. 2021	Some concerns	Some concerns	Low	Low	Low	Some concerns	
Kaur et al. 2015	Low	Low	Low	Low	Low	Low	
Mauri‐Obradors et al. 2018	Low	Low	Low	Low	Low	Low	
Mizuno et al. 2017	Low	Low	High	Low	Low	High	Data available only for 28 out of 40 subjects
Moeintaghavi et al. 2012	Low	Some concerns	Low	Low	Low	Some concerns	
Nassar et al. 2012	Some concerns	Some concerns	Low	Low	Low	Some concerns	
Nassar et al. 2014	Some concerns	Low	Low	Low	Low	Some concerns	
Pham et al. 2022	High	Low	Low	Low	Low	High	Allocation concealment, alternate allocation
Raman et al. 2014	Some concerns	Some concerns	Low	Low	Low	Some concerns	
Santos et al. 2009	Low	Some concerns	Low	Low	Low	Some concerns	
Santos et al. 2012	Some concerns	Low	Low	Low	Some concerns	Some concerns	
Santos et al. 2013	Some concerns	Low	Low	Low	Low	Some concerns	
Singh et al. 2008	Some concerns	Some concerns	Low	Low	Low	Some concerns	
Telgi et al. 2013	Some concerns	Some concerns	Low	Low	Low	Some concerns	
Toyama et al. 2014	Some concerns	Low	Low	Low	Low	Some concerns	
Wang et al. 2020	Low	Low	Low	Low	Low	Low	
Wu et al. 2015	Some concerns	Some concerns	Low	Low	Low	Some concerns	
Zhang et al. 2013	Low	Low	Low	Low	Low	Low	

### 3.2 Synthesis of the results

#### 3.2.1 OHI or no treatment versus subgingival instrumentation

A total of 11 studies compared no treatment, OHI only, or supragingival scaling with the subgingival instrumentation in patients with diabetes. In particular, five studies compared OHI to NSPT^40^, ^46^, ^50^, ^51^, ^53^ and five compared no treatment to NSPT,^34^, ^36^, ^37^, ^44^, ^49^ whereas one study compared brushing only to brushing and chlorhexidine and to NSPT plus brushing and chlorhexidine.^43^ In three studies, in the control group, the patients received also ultrasonic supragingival scaling.^48^, ^51^, ^52^ In the study published by Pham et al.,^55^ the control group received supragingival scaling and 0.05% chlorhexidine mouthwashes.

The NSPT in the study by Chen was administered following two protocols (two test groups): one with standard care with NSPT performed once (considered in the meta‐analysis) and the second with two sessions of NSPT with a 3‐month interval.^36^ The subgingival instrumentation was performed with a combination of hand curettes and sonic or ultrasonic instruments in most of the included studies, except for three papers that did not provide details.^34^, ^43^, ^50^

The authors provided no information about the severity of periodontitis.

##### Primary outcomes

The meta‐analysis at 3 months found that PPD (11 studies) and CAL (10 studies) decreased significantly more in the test group (subgingival instrumentation) than in the control one (mean 0.47 and 0.50 mm, respectively) (Table 3 and Appendix S4); the same statistical difference between test and control groups was found after 6 months for both PPD and CAL (0.56 and 0.45 mm, respectively; 7 studies analyzed for both outcomes). The subgroup analysis revealed that NSPT was effective both as compared to no treatment and as compared to OHI alone for what concerns clinical parameters (PPD and CAL).

**TABLE 3:** Results of the meta‐analysis (a positive effect size value means an advantage in the control group).

		3 months				6 months			
		Mean [95% CI] (n° of studies)	*p*	*I* ^2^	Certainty of evidence (GRADE)	Mean [95% CI] (n° of studies)	*p*	*I* ^2^	Certainty of evidence (GRADE)
OHI/No treatment versus NSPT	*PPD reduction*	0.47 [0.29, 0.65] (11) No treatment: 0.52 [0.29, 0.74] (6) OHI: 0.31 [0.23, 0.39] (3) OHI + supragingival: 0.45 [0.18, 0.72] (2)	**<0.001** **No treatment: <0.001** **OHI: <0.001** **OHI + supragingival: = 0.001**	**89%** **No treatment: 88%** **OHI: 0%** **OHI + supragingival: 0%**	Low Low Low Low	0.56 [0.28, 0.84] (7) No treatment: 0.67 [0.23, 1.11] (2) OHI: 0.31 [0.26, 0.36] (3) OHI + supragingival: 0.71 [0.34, 1.08] (2)	**<0.001** **No treatment: 0.004** **OHI: <0.001** **OHI + supragingival: <0.001**	**96%** **No treatment: 88%** **OHI: 0%** **OHI + supragingival: 66%**	Low Low Moderate Moderate
	*CAL gain*	0.50 [0.24, 0.76] (10) No treatment: 0.62 [0.34, 0.89] (5) OHI: 0.20 [0.15, 0.25] (3) OHI + Supragingival: 0.52 [0.21, 0.83] (2)	**<0.001** N**o treatment: <0.001** **OHI: <0.001** **OHI + Supragingival: = 0.001**	**91%** **No treatment: 52%** **OHI: 0%** **OHI + Supragingival: 18%**	Very low Low Low Low	0.45 [0.13, 0.77] (7) No treatment: 0.77 [0.65, 0.89] (2) OHI: 0.20 [0.12, 0.27] (3) OHI + Supragingival: 0.62 [0.15, 1.09] (2)	**0.006** **No treatment: <0.001** **OHI: <0.001** **OHI + Supragingival: = 0.001**	**93%** **No treatment: 2%** **OHI: 0%** **OHI + Supragingival: 37%**	Very low Moderate Low Low
	*BoP% reduction*	22.71 [12.97, 32.46] (6) No treatment: 28.85 [18.60, 39.11] (3) OHI: 15.90 [12.42, 19.38] (1) OHI + supragingival: 16.27 [−0.26, 32.80] (2)	**<0.001** **No treatment: <0.001** **OHI: <0.001** OHI + supragingival: 0.05	**91%** **No treatment: 82%** **OHI: N/A** OHI + supragingival: 60%	Very low Low Low Very Low	24.90 [10.48, 39.32] (6) No treatment: 28.88 [1.72, 56.04] (2) OHI: 19.88 [4.90, 34.87] (2) OHI + supragingival: 25.33 [0.58, 50.08] (2)	**<0.001** **No treatment: 0.04** **OHI: 0.009** OHI + supragingival: = 0.04	**97%** **No treatment: 97%** **OHI: 85%** OHI + supragingival: 82%	Very low Low Very low Low
	*GI reduction*	0.65 [0.33, 0.98] (7) No treatment: 0.90 [0.69, 1.10] (4) OHI: 0.37 [0.11, 0.62] (2) OHI + Supragingival: 0.30 [0.08, 0.52] (1)	**<0.001** **No treatment: <0.001** **OHI: 0.005** **OHI + supragingival: 0.007**	**95%** **No treatment: 49%** **OHI: 36%** **OHI + supragingival: N/A**	Very low Low Moderate Low	0.50 [0.11, 1.07] (4) No treatment: 1.06 [0.96, 1.16] (1) OHI: 0.30 [0.23, 0.38] (2) OHI + supragingival: 0.50 [0.28, 0.72] (1)	**<0.001** **No treatment: <0.001** **OHI: <0.001** **OHI + supragingival: <0.001**	**98%** **No treatment: N/A** **OHI: 0%** **OHI + supragingival: N/A**	Very low Low Moderate Low
	*PI reduction*	0.79 [0.39, 1.19] (6) No treatment: 0.95 [0.59, 1.30] (5) OHI + Supragingival: 0.00 [−0.20, 0.20] (1)	**<0.001** **No treatment: <0.001** OHI + supragingival: 1.00	**96%** **No treatment: 96%** **OHI + supragingival: N/A**	Very Low Low Very Low	—	—	—	—
	*PI% reduction*	17.43 [13.33, 21.54] (3) No treatment: 18.24 [4.88, 31.60] (1) OHI: 17.35 [13.03, 21.67] (2)	**<0.001** **No treatment: 0.007** **OHI: <0.001**	**0%** **No treatment: N/A** **OHI: 0%**	Low Very low Moderate	—	—	—	—
	*HbA1c reduction*	0.35 [0.07, 0.63] (11) No treatment: 0.61 [0.51, 0.72] (6) OHI: −0.01 [−0.10, 0.08] (3) OHI + Supragingival: 0.10 [−1.03, 1.23] (2)	**0.01** **No treatment: <0.001** OHI: 0.80 OHI + supragingival: 0.86	**89%** No treatment: 0% OHI: 0% OHI + supragingival: N/A	Very low Moderate Very low Very low	0.35 [0.04, 0.67] (8) No treatment: 0.80 [0.13, 1.47] (2) OHI: 0.12 [−0.222, 0.46] (3) OHI + Supragingival: 0.42 [0.06, 0.79] (3)	**0.03** **No treatment: 0.02** OHI: 0.49 **OHI + supragingival: 0.02 (2)**	**88%** **No treatment: 65%** OHI: 89% **OHI + supragingival: 0%**	Very low Low Very low Low
NSPT with FMD protocol versus NSPT with non‐FMD protocol	*PPD reduction*	−0.09 [−0.41, 0.23] (6)	0.57	52%	Very low	0.05 [−0.07, 0.17] (6)	0.40	0%	Very low
	*CAL gain*	0.01 [−0.34, 0.36] (6)	0.95	28%	Very low	0.03 [−0.25, 0.31] (6)	0.83	0%	Very low
	*BoP% reduction*	−5.01 [−10.55, 0.53] (7)	0.08	57%	Very low	−7.63 [−17.67, 2.41] (5)	0.14	84%	Very low
	*HbA1c reduction*	−0.32 [−1.05, 0.41] (6)	0.39	0%	Very low	−0.37 [−1.24, 0.50] (5)	0.41	46%	Very low
	*% sites with PPD ≥5 mm*	−5.80 [−13.49, 1.89] (3)	0.14	41%	Very Low	—	—	—	—

Abbreviations: 95% CI, 95% confidence interval; BoP%, bleeding on probing %; CAL, clinical attachment level; FMD, full‐mouth disinfection; GI, Gingival index; NSPT, nonsurgical periodontal treatment; OHI, oral hygiene instructions; PI%, Plaque index %; PI, Plaque index; PPD, pocket/periodontal probing depth.

None of the studies included in the meta‐analysis reported on the proportions of pocket closure after treatment.

The study by Artese and coworkers,^48^ not included in the meta‐analysis, reported on the decrease in the proportion of sites with PPD higher than 4 mm, with a significant difference between groups regarding the decrease of sites with PPD between 4 and 6 mm and not for sites with PPD equal or more than 7 mm.

The sensitivity analysis, by removing studies at high risk of bias, revealed evidence of a significant difference between the test and control group for PPD (0.48 mm, 95% CI 0.28–0.68 mm at 3 months; 0.56 mm, 95% CI 0.26–0.87 mm at 6 months) and for CAL (0.51 mm, 95% CI 0.21–0.82 mm at 3 months; 0.40 mm, 95% CI 0.02–0.79 mm at 6 months) (Appendix S5).

The analysis without the studies using CHX found evidence of a significant difference between the two groups for PPD (0.47 mm, 95% CI 0.22–0.71 mm at 3 months; 0.56 mm, 95% CI 0.26–0.87 mm at 6 months) and for CAL (0.49 mm, 95% CI 0.19–0.79 mm at 3 months; 0.39 mm, 95% CI 0.03–0.74 mm at 6 months) (Appendix S5).

##### Secondary outcomes

The meta‐analysis showed a significant difference in the decrease of the periodontal secondary outcomes (BoP%, GI, PI, and PI%) at 3 months, and the difference was still statistically significant after 6 months for BoP% and GI (Table 3). Considering HbA1c changes, the parameter improved significantly in the NSPT group after 3 and 6 months (the improvement was significant as compared to no treatment, as well as to OHI and supragingival scaling, but not as compared to the OHI subgroup).

None of the studies included in the meta‐analysis reported on PROMs.

The sensitivity analysis removing studies at high risk of bias found evidence of a significant difference between the test and control group for BoP% (25.20, 95% CI: 13.34–37.07 at 3 months; 24.74, 95% CI 6.68–42.81 at 6 months) and for PI (0.95, 95% CI 0.59–1.30 at 3 months).

The analysis after removing studies using CHX revealed a significant difference between the two groups for BoP% reduction (22.41, 95% CI 11.47–33.35 at 3 months; 22.52, 95% CI 6.30–38.73 at 6 months), for GI reduction (0.75, 95% CI 0.35–1.15 at 3 months; 0.62, 95% CI 0.00–1.23 at 6 months), for PI reduction (0.99, 95% CI 0.49–1.49 at 3 months), for PI% reduction (18.02, 95% CI 13.75–22.30 at 3 months), and for HbA1c reduction (0.31, 95% CI 0.01–0.60 at 3 months; 0.35, 95% CI 0.04–0.67 at 6 months) (Appendix S5).

No data on site‐specific response to subgingival instrumentation (in horizontal defects, intrabony defects, and furcations) were reported by the included studies.

##### Certainty of evidence (GRADE)

Regarding the certainty of evidence it was classified as “Very low” or “Low” for most of the outcomes and moderate for PPD reduction (6 months) when controls are OHI groups and OHI + supragingival scaling, CAL reduction (6 months) with negative controls, GI reduction (3 months and 6 months) when controls are OHI groups, PI% reduction (3 months) when controls are OHI groups, and HbA1c% reduction (3 months) with negative controls (Appendix S6).

#### 3.2.2 NSPT with full‐mouth protocol versus quadrant protocol

A total of seven studies explored the clinical efficacy of a full‐mouth NSPT protocol versus a quadrant NSPT protocol.^35^, ^38^, ^39^, ^41^, ^42^, ^47^, ^56^ Different protocols were applied for the use of antimicrobials, varying from no antimicrobial therapy,^35^, ^39^, ^56^ to the use of chlorhexidine during the sub‐gingival instrumentation session,^41^, ^47^ and to the administration of CHX mouth rinses for 60 days.^42^ The authors provided no information about the severity of periodontitis.

##### Primary outcomes

The meta‐analysis performed on seven studies did not show any significant difference between groups for the primary outcomes (Table 3). The study by Graziani and coworkers^56^ provided also results about the number of sites with PPD equal or higher than 5 mm, finding no differences between the two groups.

The sensitivity analysis without the studies using CHX found no evidence of a significant difference between the two groups for PPD or CAL (Appendix S5).

##### Secondary outcomes

A lack of statistically significant difference was also found for the secondary outcomes. Moreover, Graziani et al.^56^ reported on vital signs and serum markers, and on patient‐reported outcomes, without revealing the presence of any difference between groups. No data on site‐specific response to subgingival instrumentation (in horizontal defects, intrabony defects, and furcations) were reported by the included studies.

The sensitivity analysis found no evidence of a difference between the test and control group for all outcomes (Appendix S5).

##### Certainty of evidence (GRADE)

The certainty of evidence was classified as “Very low” for all the outcomes (Appendix S6).

#### 3.2.3 NSPT versus NSPT plus OHI performed with the aid of community health workers

One study compared NSPT to NSPT plus OHI performed with the aid of community health workers (CHW) who are people, in Taiwan, who have “in‐depth knowledge of the communities they serve.”^54^ The sample was made of a total of 76 patients (37 in the control group and 39 in the test group) that were followed up for 6 months. The authors provided no information about the severity of periodontitis.

##### Primary outcomes

The study found a positive effect of CHW aid in terms of clinical results, although the effect size was negligible (PPD difference between groups after 6 months was 0.1 mm, 95% CI: −0.36, 0.11; CAL gain difference between groups after 6 months was 0.1 mm, 95% CI: −0.37, 0.16).

##### Secondary outcomes

Similarly, small differences were observed for secondary outcomes favoring the experimental group for BOP% at 6 months (with a difference of −0.3, 95% CI: −12.04, 11.35) and the control group for GI and PI at 6 months (differences of 0.2, 95% CI: −0.17, 0.51, and 0.2, 95% CI: −0.14, 0.58, respectively). No difference in the reduction of HbA1c levels was observed between groups.

#### 3.2.4 Periodontal treatment in subjects with controlled versus uncontrolled diabetes

One study compared treatment versus no treatment in subjects with controlled (HbA1c < 7%) or uncontrolled diabetes, in India.^49^ A total of 100 subjects were included (48 with good glycemic control, 23 treated and 25 not treated; 52 with poor glycemic control, 27 treated and 25 not treated), and they were followed up for 6 months. The authors gave no information about the severity of the periodontitis.

##### Primary outcomes

Considering the patients that received nonsurgical treatment, in the poor glycemic control group (*n* = 27) PPD decreased by 0.79 + − 0.26 mm after 6 months while in the good glycemic control group (*n* = 23) it decreased by 0.84 + − 0.31, without any statistical evidence of a difference. CAL gain was 0.71 + − 0.38 mm in the poor glycemic control group and 0.72 + − 0.34 in the good glycemic control group. No evidence of a difference was found also for the changes in pocket depth proportions in the two groups.

##### Secondary outcomes

In treatment groups, no significant differences could be found between the poor glycemic control group (*n* = 27) and the good glycemic control group (*n* = 23) regarding GI, PI, and BOP% at 6 months, although all the parameters decreased significantly from baseline. HbA1c decreased by 0.16 + − 0.32 in the good glycemic control group and by 1.49 + − 0.98 in the poor glycemic control group, without any evidence of a statistical difference between the two groups.

No data on site‐specific response to subgingival instrumentation (in horizontal defects, intrabony defects, and furcations) were reported by the included studies.

## 4 DISCUSSION

The present systematic review indicated that in type 2 diabetic patients NSPT performed through subgingival scaling is, as expected, more effective than oral hygiene alone or associated with just supragingival scaling with regards to all periodontal clinical outcomes, including a small but statistically significant effect on HbA1c. Full‐mouth treatment protocols and other quadrant‐based protocols seem equally effective. However, the quality of the available evidence, as measured using the GRADE tools, for the considered comparisons and outcomes is generally low/very low.

The clinical significance of the results we obtained should be considered in the context of the existing literature, with consideration of the effect sizes. The recent systematic review by Suvan and coworkers evaluated the efficacy of subgingival instrumentation as compared to supragingival/no instrumentation for the treatment of periodontitis or no treatment.^18^ The authors found only one study comparing scaling and root planing and no treatment, revealing a significant benefit in the first group in terms of the proportion of closed pockets.^57^ However, changes in clinical parameters were not reported, hence comparison with our results could not be done. In the field of periodontal therapy, periodontitis requires oral hygiene instructions, supragingival scaling, and subgingival nonsurgical treatment, and this is widely accepted by the scientific community.^17^, ^58^ For this reason, in clinical research, the comparison between treatment and no treatment is nowadays considered unethical although, in the included studies, the period of no treatment was no longer than 3–6 months. Our data are in line with the review by Suvan et al.,^18^ on healthy subjects, and the recent Cochrane systematic review,^20^ on subjects with diabetes, since subgingival instrumentation led to significant improvements both in PPD and CAL, as well as in the HbA1c level, compared to no treatment. As in the previously cited reviews, the present analysis could not provide any information about the effect of the severity of periodontal diseases on the outcomes of the treatment in a cohort of patients with diabetes and, in the included studies, the older classifications of the periodontal diseases were adopted, thus limiting the comparability among studies.

Another recent systematic review of the literature by Oliveira and coworkers included 11 studies in the meta‐analysis, comparing subgingival therapy (with or without access surgery) to non‐active therapy.^59^ Differently from the present review, the authors pooled the data belonging to different treatment protocols, without subgroup analysis, finding a significantly higher reduction of HbA1c in the treatment group than control group (Delta = 0.29 CI95%: 0.10–0.47). The data were in line with the one resulting from our meta‐analysis, even though performing the subgroup analysis allowed us to speculate that the effect on HbA1c was significant only when comparing treatment versus no treatment. The effect of supragingival scaling and oral hygiene on HbA1c should be further explored in subjects without periodontitis. Results from a recent cohort study found that, in the medium and long‐term, the difference in HbA1c between treated and untreated subjects may become negligible.^60^

One systematic review and meta‐analysis, published in 2024, examined the effects of nonsurgical periodontal therapy in subjects with systemic comorbidities, including diabetes. The authors included 21 trials performed in diabetic patients, pooling together, in the meta‐analysis, different treatment options and comparators.^23^ The results of the study, regarding clinical outcomes, are comparable to those found in our meta‐analysis about the comparison between treatment and no treatment.^23^

The present systematic review also focused on comparing the efficacy of different subgingival treatment protocols in subjects affected by diabetes. As reported in many systematic reviews of the literature, in healthy subjects there is no evidence that full‐mouth treatment protocol is more effective than quadrant/sextant‐based protocols in terms of clinical outcomes.^18^, ^61^ Considering the underlying diabetic condition and the concomitant use of diabetic medications which make diabetic patients more susceptible to infections, it might be speculated that a full‐mouth approach could present some advantages over a quadrant approach. In particular, a full‐mouth protocol would decrease the number of visits and would lead to an abrupt reduction in bacterial infection, limiting the risk of re‐infection of the already instrumented sites from sites not yet treated and/or other intraoral niches. However, our results indicated that the two treatment protocols were equally effective for all the outcomes considered. In other words, the presence of diabetes does not seem to influence the relative response to non‐surgical periodontal treatment, thus allowing the application of different protocols. In some way, we found a confirmation of what was reported in another systematic review of the literature about laser applications as an adjunct to nonsurgical treatment in subjects with diabetes and periodontitis.^62^ The most recent systematic review about the treatment of diabetic subjects, as stated above, did not distinguish among treatment protocols.^23^

Our review has the merit to have combined the meta‐analyses with a transparent framework (GRADE) that provides a systematic approach making clinical practice recommendations. Such an approach is designed also to give information about the quality of the evidence that resulted from a meta‐analysis, being of help in decision‐making processes.^63^ The GRADE approach is used for clinical guidelines and best/good practice statements^64^ and, to the best of our knowledge, no systematic reviews exploring the topic of the present study were published adopting such an approach. In the context of systematic reviews, the quality of evidence is defined as the “extent of confidence that an estimate of effect is correct.”^65^ Since overall, the quality of evidence was low/very low, caution should be adopted in the generalization of the present results. The main reasons for downgrading the quality of evidence were the presence of studies with a high risk of bias, heterogeneity, and imprecision (wide confidence intervals).

Finally, the validity of the results of this review should be weighted also in consideration of its limitations. First, limited data could be extrapolated about pocket closure, which should be considered as the endpoint of therapy and mean full‐mouth PPD and CAL values changes may not be reliable outcomes to capture treatment response to NSPT.^66^, ^67^ Moreover, no studies reported on PROMs, which are key aspects to take into consideration when comparing different treatment protocols, in particular in subjects with systemic diseases.^68^ Another limitation is represented by the heterogeneity of the study protocols and certain heterogeneity in the definition of periodontitis and diabetes reported by the included studies, although we tried to account for this by performing subgroup analysis and sensitivity analysis whenever possible, which represents an important strength of the present review. We did not have the possibility of performing a sub‐analysis based on age groups since we have no data from the studies. Then, we have no or few information about the severity of periodontitis and the effect of the level of glycemic control on the response to periodontal treatment.

Despite the limitations cited above, the present review has the strength of having addressed the quality of evidence evaluation and focusing on both periodontal outcomes and glycemic control, providing a comprehensive review of both aspects.

The clinical relevance of the results is represented by the evidence of no differences in different treatment options considering both clinical and glycemic control outcomes. In other words, in subjects with diabetes, nonsurgical periodontal treatments resulted in significant improvement of the desired outcomes, independently from the characteristics of the treatment themselves.

## 5 CONCLUSIONS

### 5.1 Implications for clinical practice

Considering the limits of our investigation we can conclude that:

1. In subjects with diabetes, NSPT is effective and leads to an improvement in periodontal clinical parameters similar to what is expected in healthy individuals, and it improves glycemic control in the short term (3–6 months) as compared to treatments not including subgingival instrumentation.
2. In subjects with diabetes, NSPT can be performed both through a full‐mouth protocol or through a quadrant/sextant‐based protocol, with comparable improvements in clinical parameters.

Based on the data extracted from the included studies, we can speculate that nonsurgical periodontal treatment protocols work similarly in patients with uncontrolled diabetes as well as in subjects with good glycemic control.

### 5.2 Implications for research

More studies, comparing different nonsurgical protocols and evaluating the impact of the maintenance phase, with longer follow‐ups (1 year and more), could be helpful for better evaluating the stability of the results obtained in the short term. While the clinical efficacy of the different clinical protocols assessed in this review was the same, it would be interesting in the future to assess the impact that different protocols may have on other relevant outcomes, such as PROMs and the risk of adverse events.

## CONFLICT OF INTEREST STATEMENT

None.

## Supporting information


Appendixes.

## Data Availability

Data will be available on request.
