# Supplementary material for: Efficacy of different protocols of non‐surgical periodontal therapy in patients with type 2 diabetes: A systematic review and meta‐analysis
Source: J Periodontal Res. 2024 Sep 29;60(5):417–37. doi: 10.1111/jre.13327 (PMC12186468; doi:10.1111/jre.13327)
Supplement: Supplementary file 1 — Appendixes. [file JRE-60-417-s001.docx]

**Appendix S1**

**Search strategy**

**MEDLINE via OVID (1946 to 17/07/2023): 3958**

|  | MeSH term | Free-Text search |
| --- | --- | --- |
| Population | Exp Periodontal Diseases OR alveolar bone loss/  AND  Exp diabetes mellitus, Type 2 OR exp hyperglycemia | Periodontit* OR Parodontos$s OR periodontal disease OR pyorrhea OR Pericementit* OR gum disease OR (Periodont* ADJ2 pocket*) OR (Periodont* ADJ2 defect*) OR (Periodont* ADJ2 atroph*) OR (periodontal attachment ADJ2 loss) OR (periodontal bone ADJ2 loss*) OR (periodontal ADJ2 resorption*) OR furcation OR (alveolar bone ADJ2 loss)  AND  diabet* or DM2 OR NIDDM or hyperglyc* |

The limitation to human studies was performed following the double negation strategy suggested by the Cochrane handbook, i.e. combining the results with NOT (exp animals/ not humans.sh.).

In addition, the following filters were applied:

Fillter to exclude systematic reviews:

NOT (((systematic OR state-of-the-art OR scoping OR literature OR umbrella) ADJ (review* OR overview* OR assessment*)) OR "review* of reviews" OR meta-analy* OR metaanaly* OR ((systematic OR evidence) ADJ1 assess*) OR "research evidence" OR metasynthe* OR meta-synthe*).tw. OR systematic review/ OR "systematic review (topic)"/ OR meta analysis/ OR "meta analysis (topic)"/

Filter to exclude case reports and other non-relevant pubblications:

NOT (letter/ OR editorial/ OR news/ OR exp historical article/ OR anecdotes as topic/) OR (letter OR comment*.ti) OR case reports/

Filter to exclude guidelines:

(clinical adj3 pathway).ti,ab,kw. or (clinical adj3 pathways).ti,ab,kw. or (practice adj3 parameter).ti,ab,kw. or (practice adj3 parameters).ti,ab,kw. or algorithms/ or care pathway.ti,ab,kw. or care pathways.ti,ab,kw. or clinical protocols/ or Consensus/ or Consensus Development Conference.pt. or Consensus Development Conference, NIH.pt. or Consensus Development Conferences as Topic/ or Consensus Development Conferences, NIH as Topic/or critical pathway/ or guidance.ti,ab. or guideline*.ti. or guidelines as topic/ or practice guidelines as topic/ or Health Planning Guidelines/ or practice guideline/

**Cochrane library via CENTER (17/07/2023): 645 (and 73 systematic reviews)**

|  | MeSH term | Free-Text search |
| --- | --- | --- |
| Population | Exp Periodontal Diseases OR exp alveolar bone loss  AND  Exp diabetes mellitus, Type 2 OR exp hyperglycemia | Periodontit* OR Parodontos$s OR periodontal disease OR pyorrhea OR Pericementit* OR gum disease OR (Periodont* NEAR/2 pocket*) OR (Periodont* NEAR/2 defect*) OR (Periodont* NEAR/2 atroph*) OR (periodontal attachment NEAR/2 loss) OR (periodontal bone NEAR/2 loss*) OR (periodontal NEAR/2 resorption*) OR furcation OR (alveolar bone NEAR/2 loss)  AND  diabet* or DM2 OR NIDDM or hyperglyc* |

**EMBASE (17/07/2023): 2986**

|  | EMTREE terms | Free-Text search |
| --- | --- | --- |
| **Population** | Exp Periodontal Disease OR alveolar bone loss  AND  Exp non insulin dependent diabetes mellitus OR exp hyperglycemia | Periodontit* OR Parodontos$s OR periodontal disease OR pyorrhea OR Pericementit* OR gum disease OR (Periodont* ADJ2 pocket*) OR (Periodont* NEAR/2 defect*) OR (Periodont* NEAR/2 atroph*) OR (periodontal attachment NEAR/2 loss) OR (periodontal bone NEAR/2 loss*) OR (periodontal NEAR/2 resorption*) OR furcation OR (alveolar bone NEAR/2 loss)  AND  diabet* or DM2 OR NIDDM or hyperglyc* |

The results were limited to human studies, clinical studies, articles and articles in press with the dedicated EMBASE limits.

**Appendix S2**

For each presented outcome, the difference between baseline and follow-up values were extracted (with specific error measure such as standard deviation (SD) or standard error (SE) or variance). When such parameter was not presented, it was computed as the difference between baseline and follow-up values. In these cases, following the instructions of the Cochrane Handbook for Systematic Reviews when SDs of changes values were not presented and they were not provided by authors after contacting them by email, they were computed as follows: i) if similar studies were present (similar treatment, similar population, similar sample size), SD was imputed taking the value of the other study; ii) when *P* value is presented SD was computed by using T tables for retrieving SEs; iii) when *P* value is presented as a limit (e.g. < 0.05) a conservative value of *P* (e.g. 0.05 in case of < 0.05) was considered for computing SE as described before; iv) if *P* value was not present SDs of change values was imputed by using the following formula ^24,69,70^:

$$SDcv= \sqrt{{SDbaseline}^{2}+ {SDfinal}^{2}-(2*CORR*SDbaseline*SD final)}$$

being CORR the correlation coefficient, that could be imputed from similar studies if present, or it was assumed conservatively to be 0.2. For each measure, pooled estimate of 95% CI was calculated.

**Appendix S3**

**Risk of Bias summary**

**
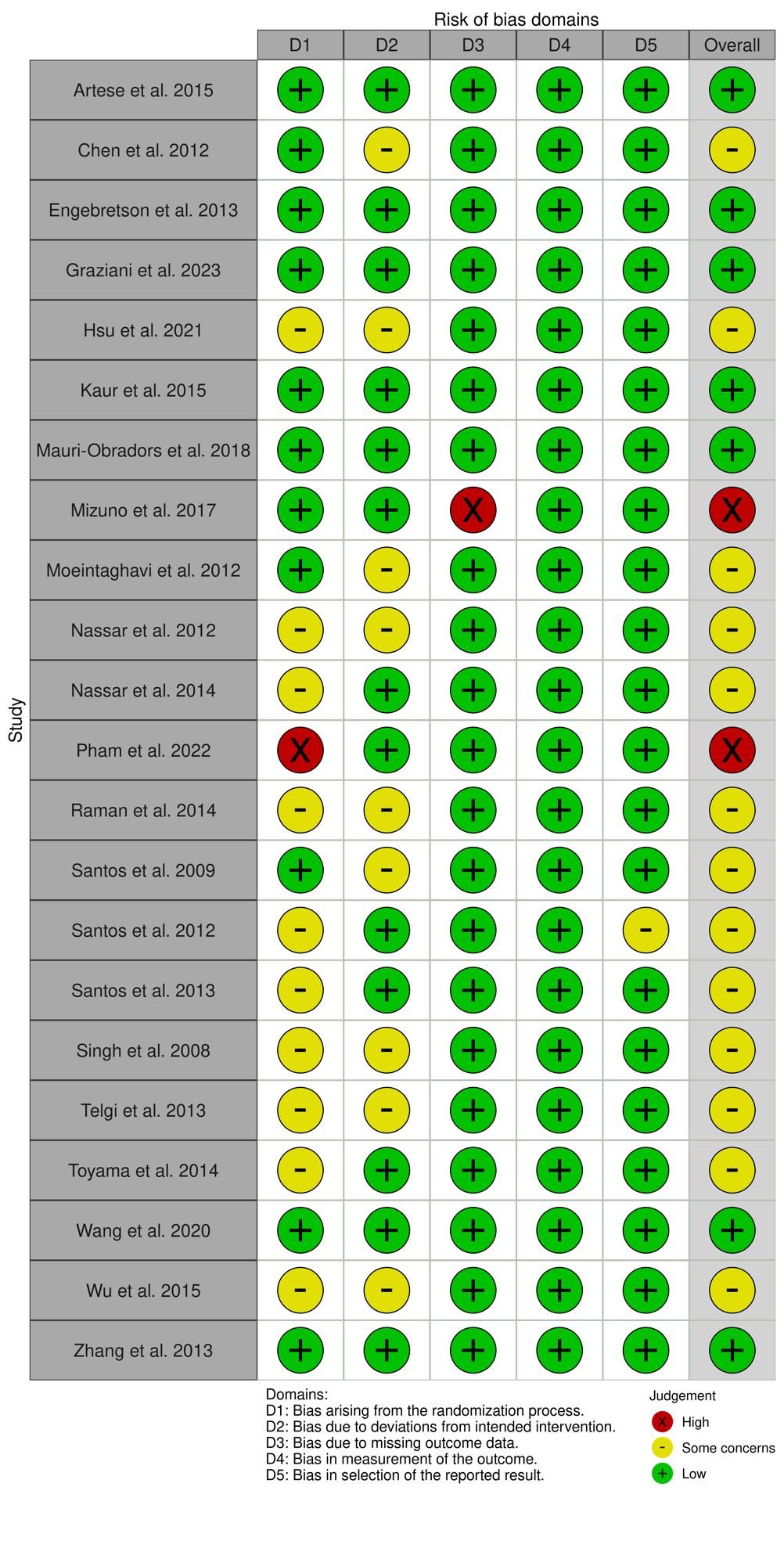
**

**
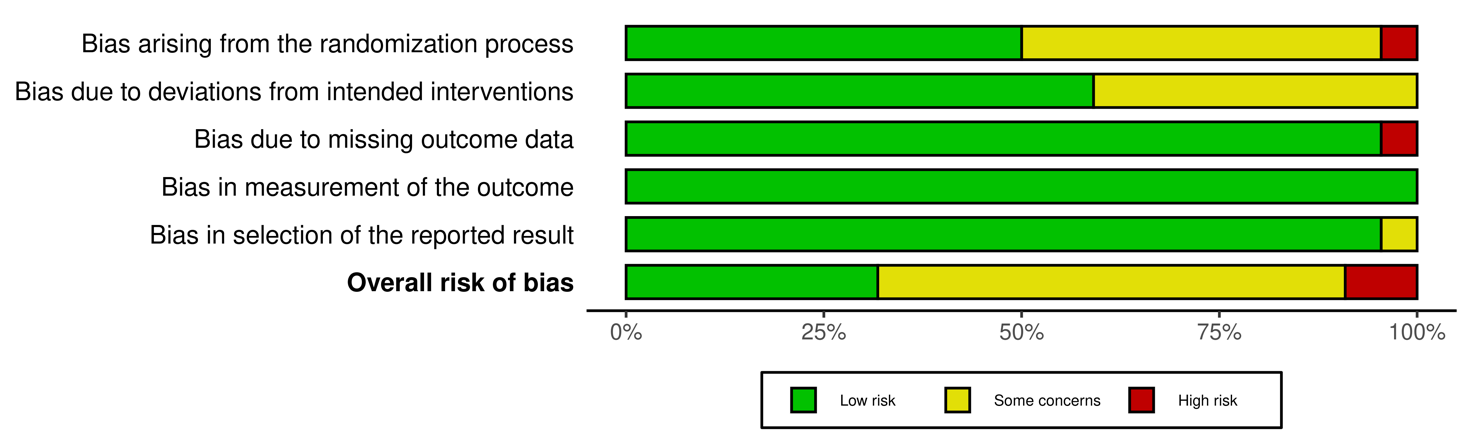
**

**Appendix S4**

**Forest plots**

OHI / No treatment vs NSPT (PPD reduction – 3 months)


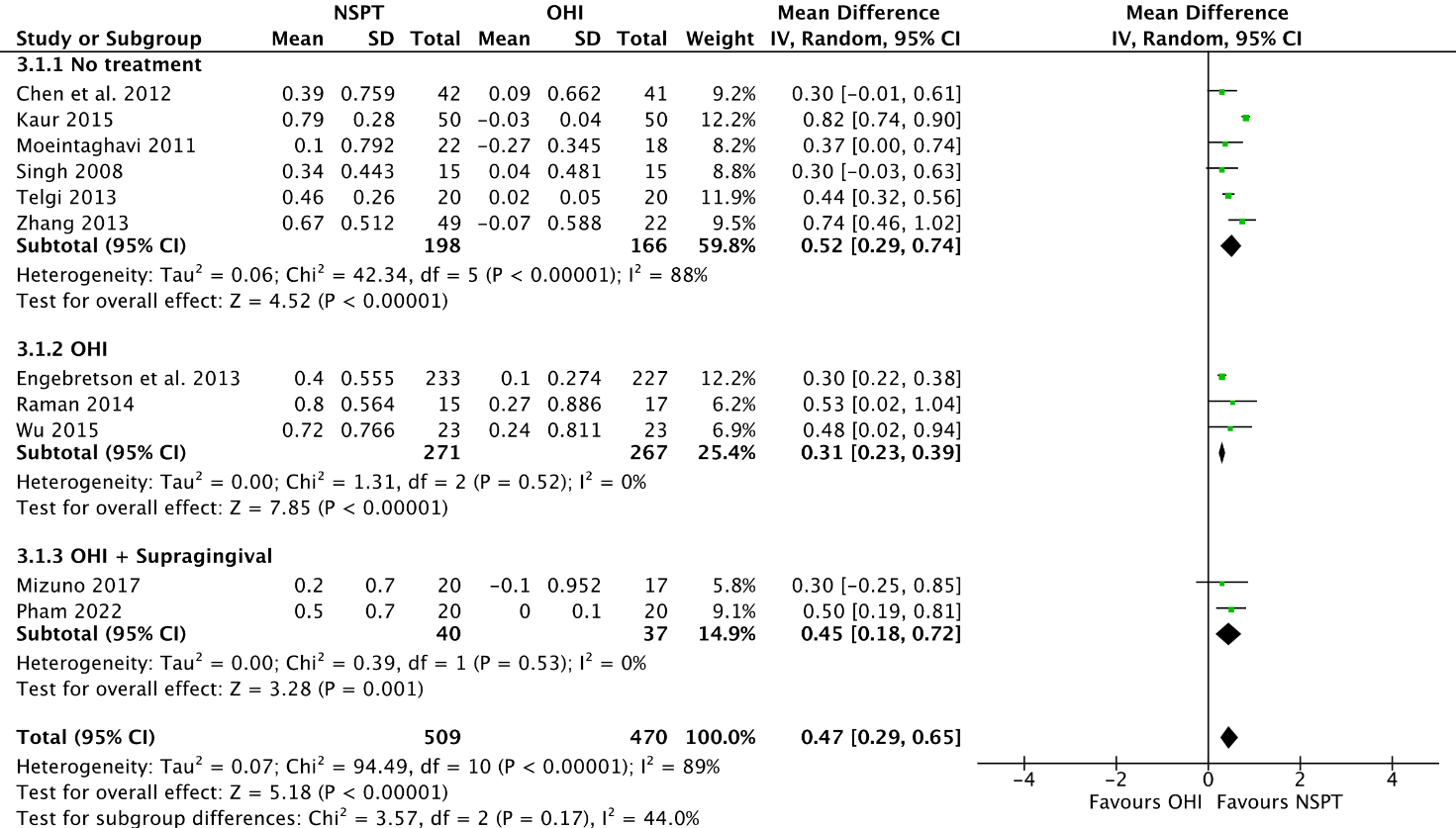


OHI / No treatment vs NSPT (PPD reduction – 6 months)


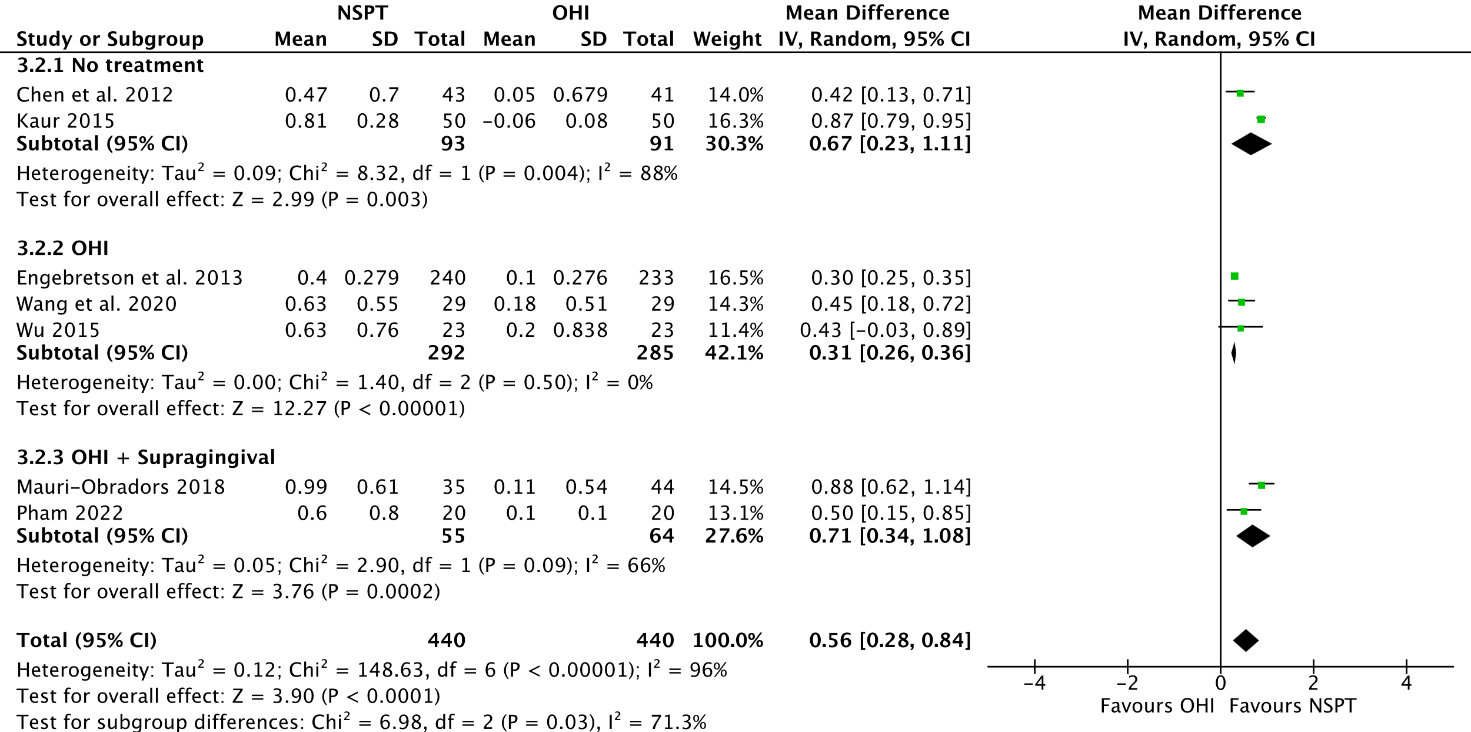


OHI / No treatment vs NSPT (CAL gain – 3 months)


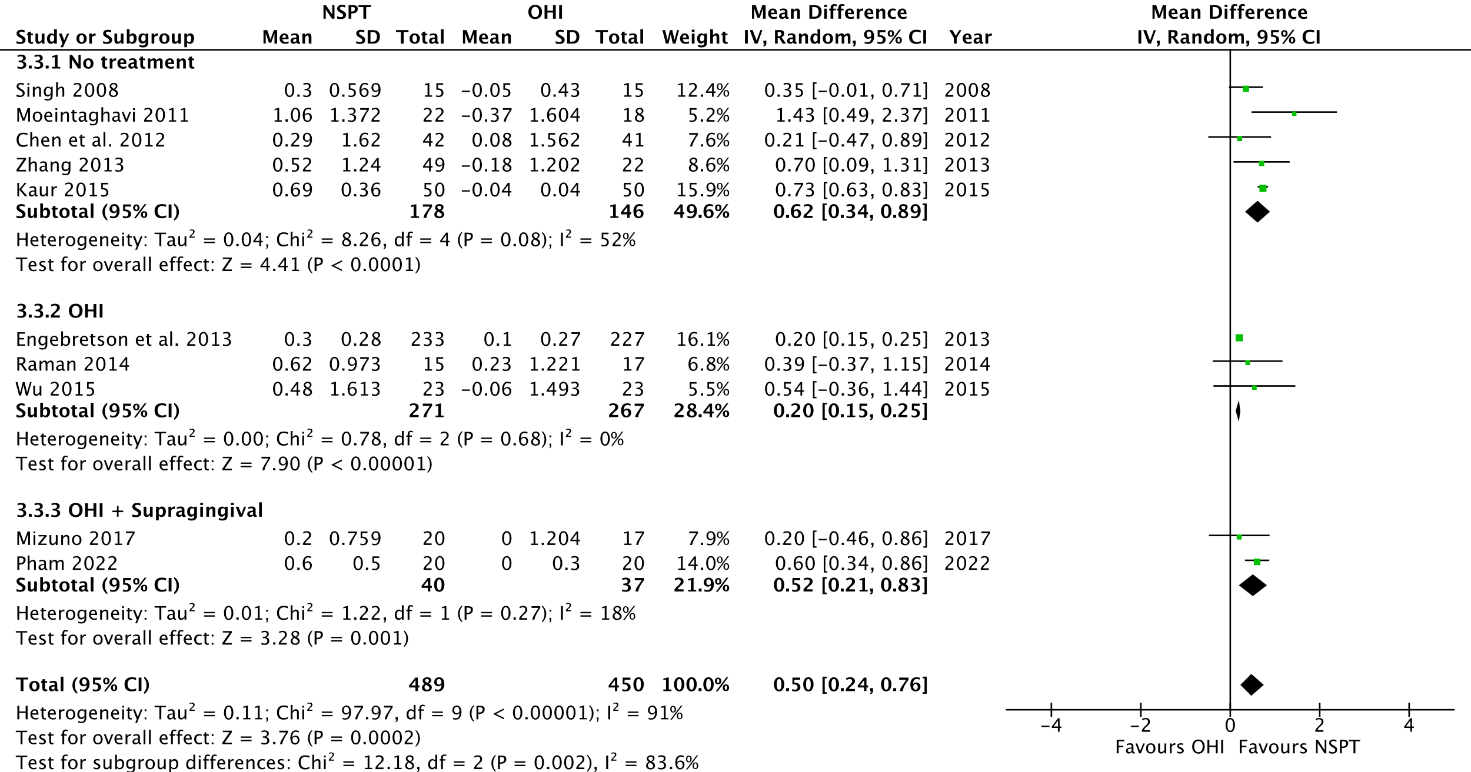


OHI / No treatment vs NSPT (CAL gain – 6 months)


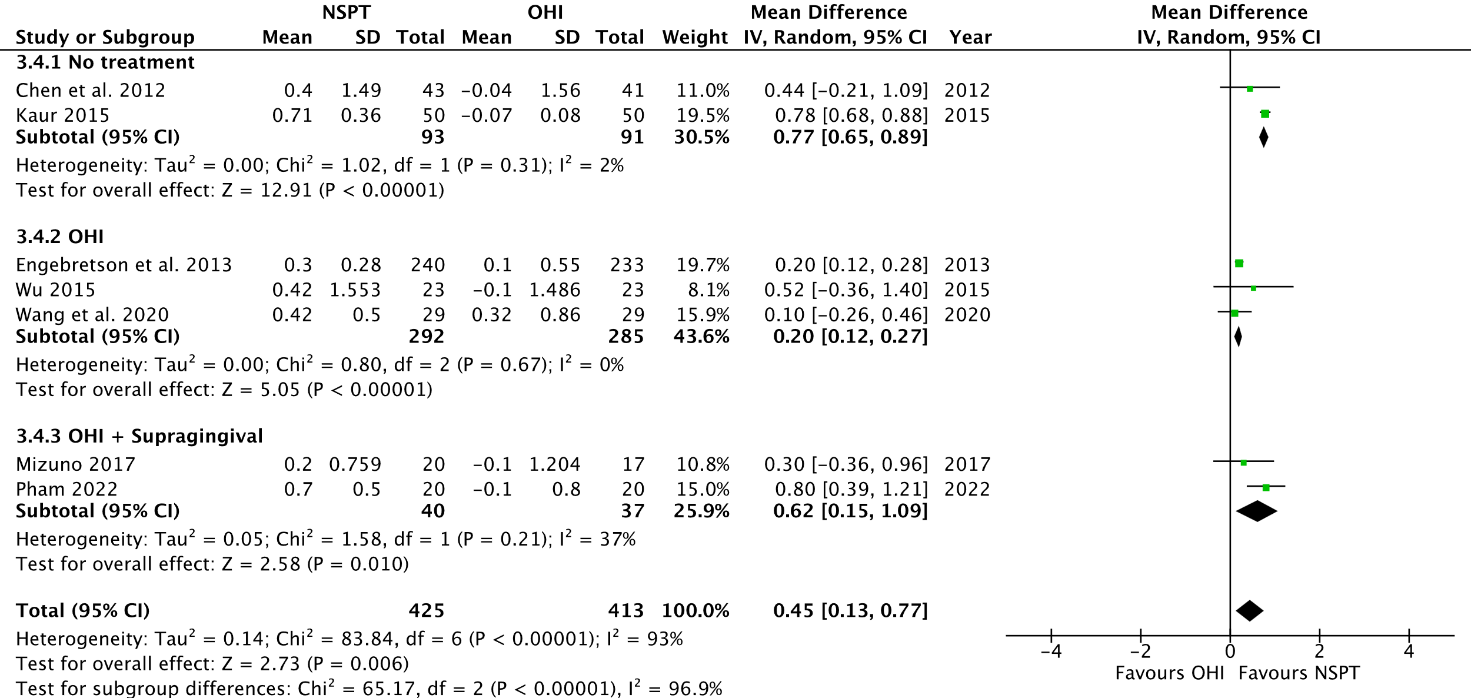


OHI / No treatment vs NSPT (BoP% reduction – 3 months)


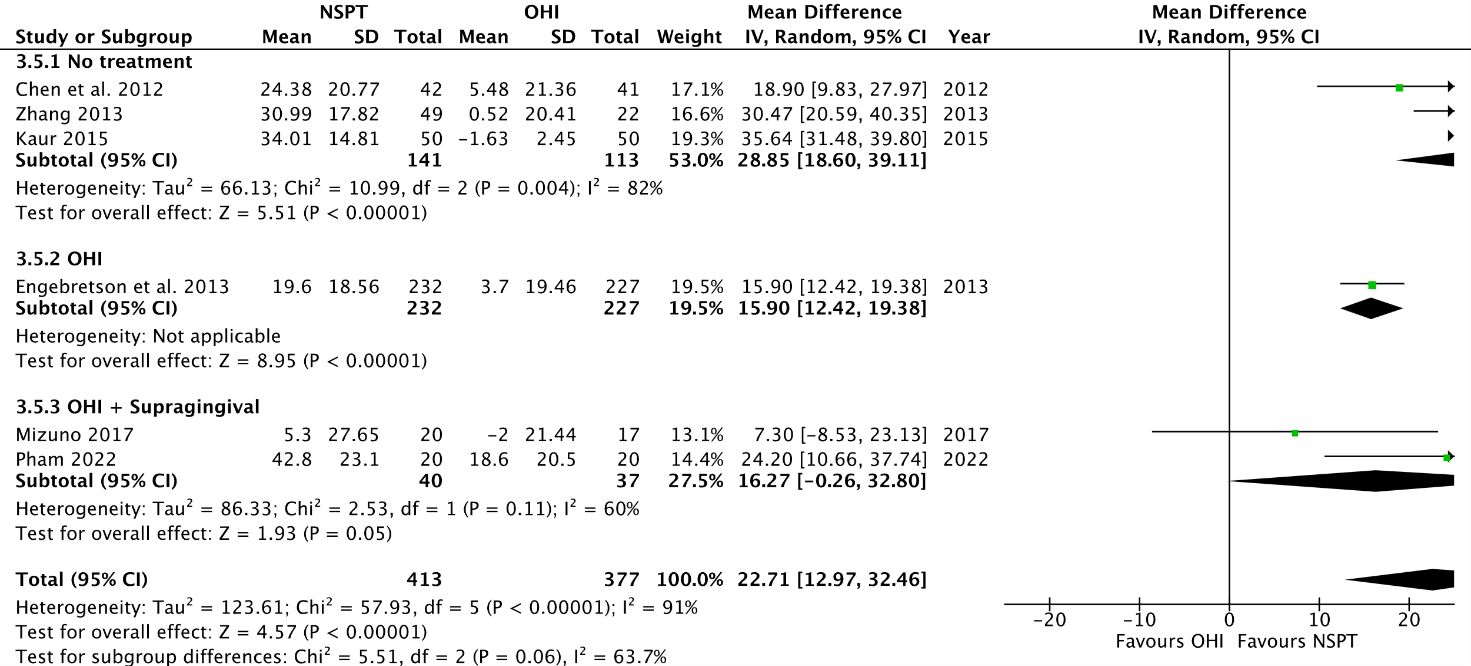


OHI / No treatment vs NSPT (BoP% reduction – 6 months)


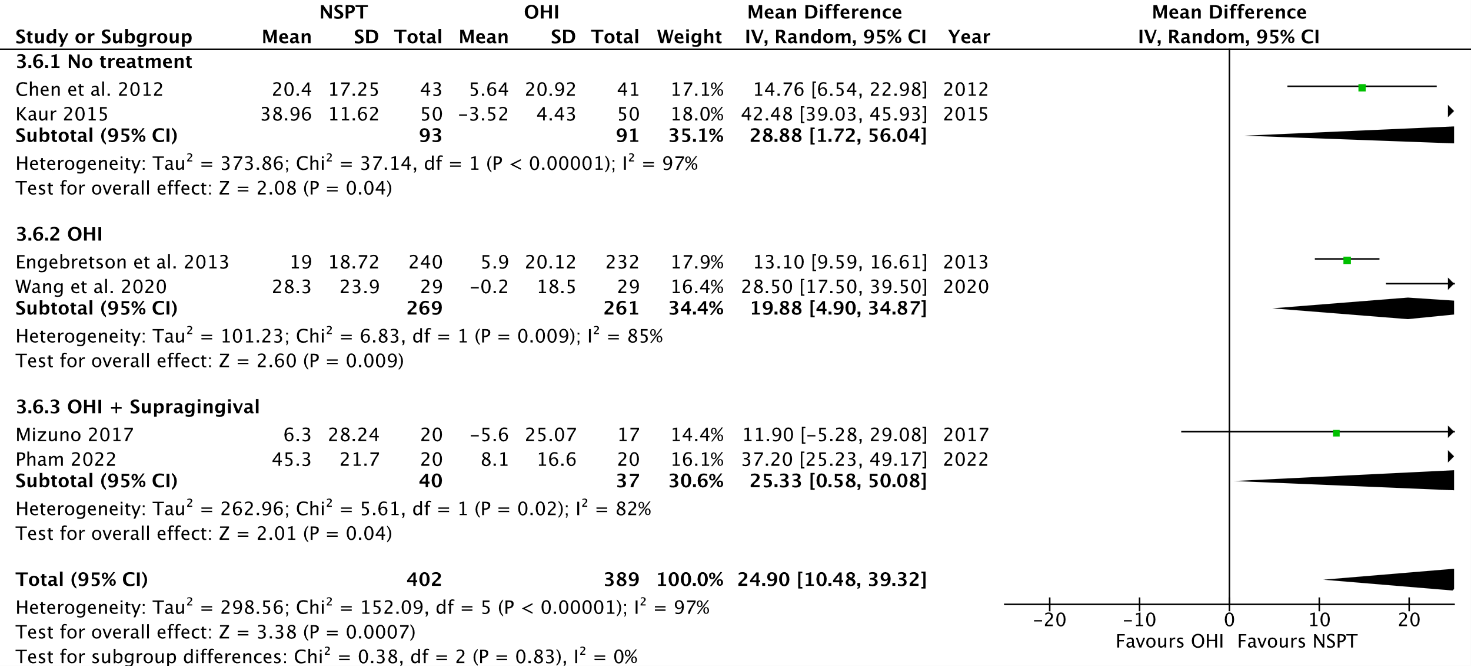


OHI / No treatment vs NSPT (GI reduction – 3 months)


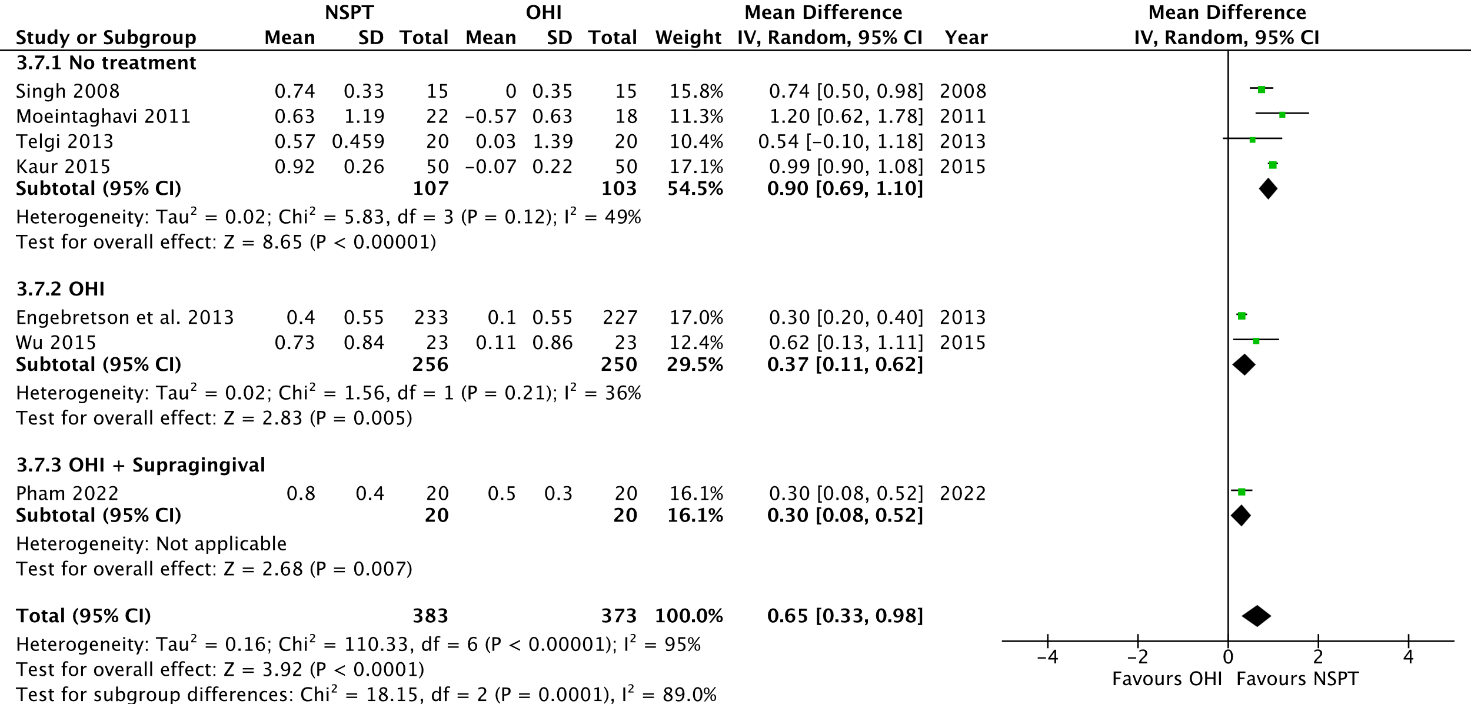


OHI / No treatment vs NSPT (GI reduction – 6 months)


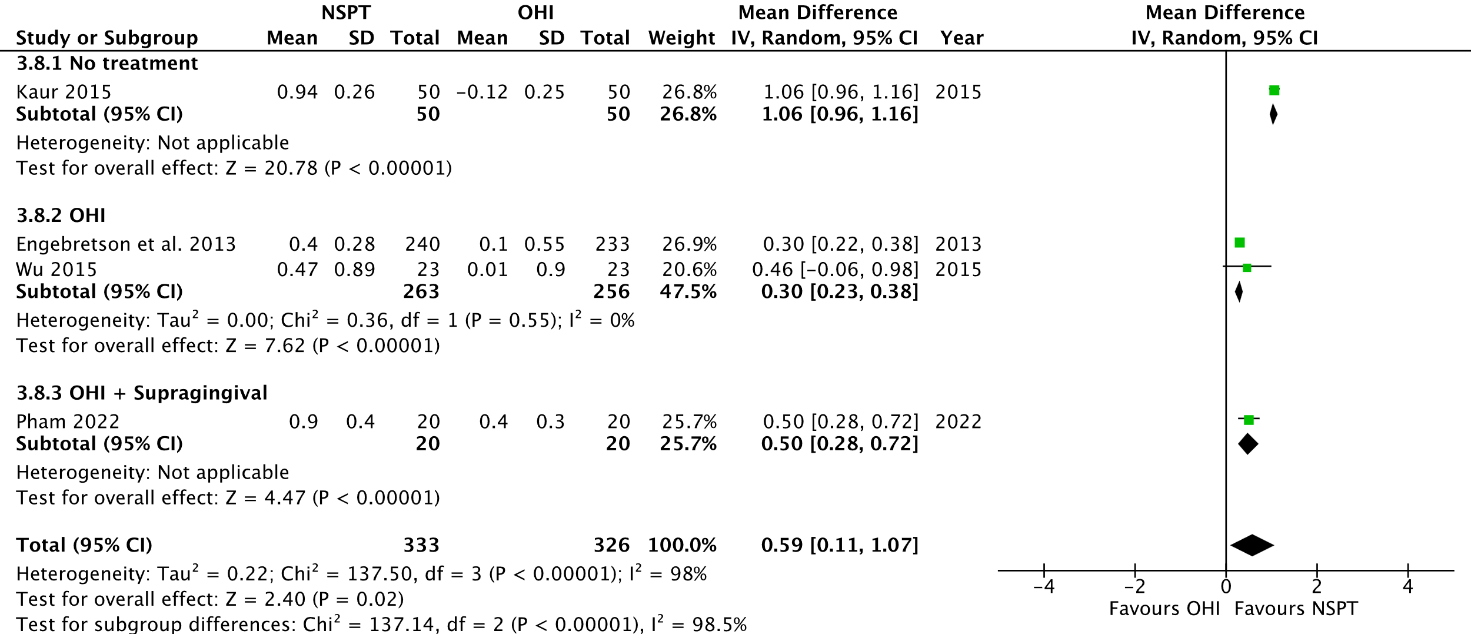


OHI / No treatment vs NSPT (PI reduction – 3 months)


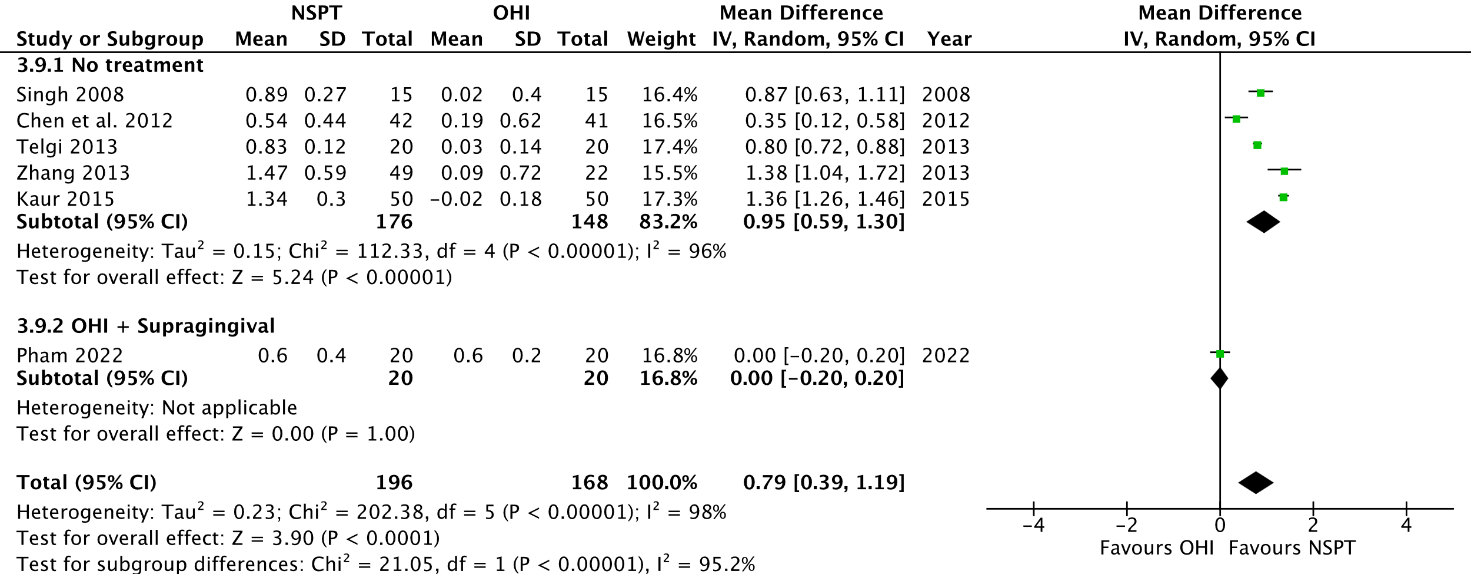


OHI / No treatment vs NSPT (PI% reduction – 3 months)


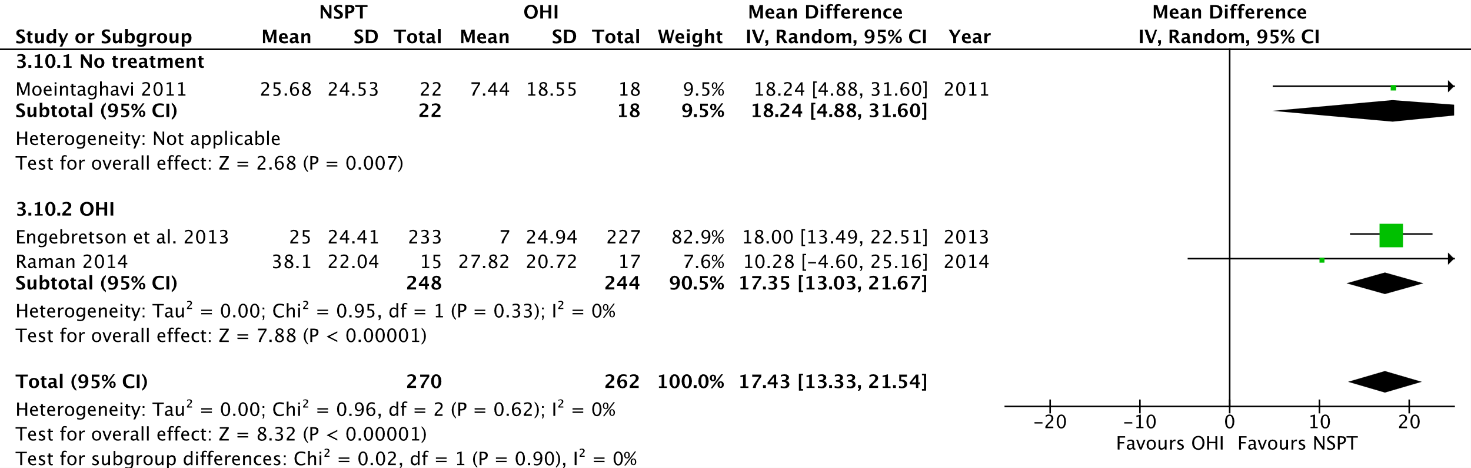


OHI / No treatment vs NSPT (HbA1c% reduction – 3 months)


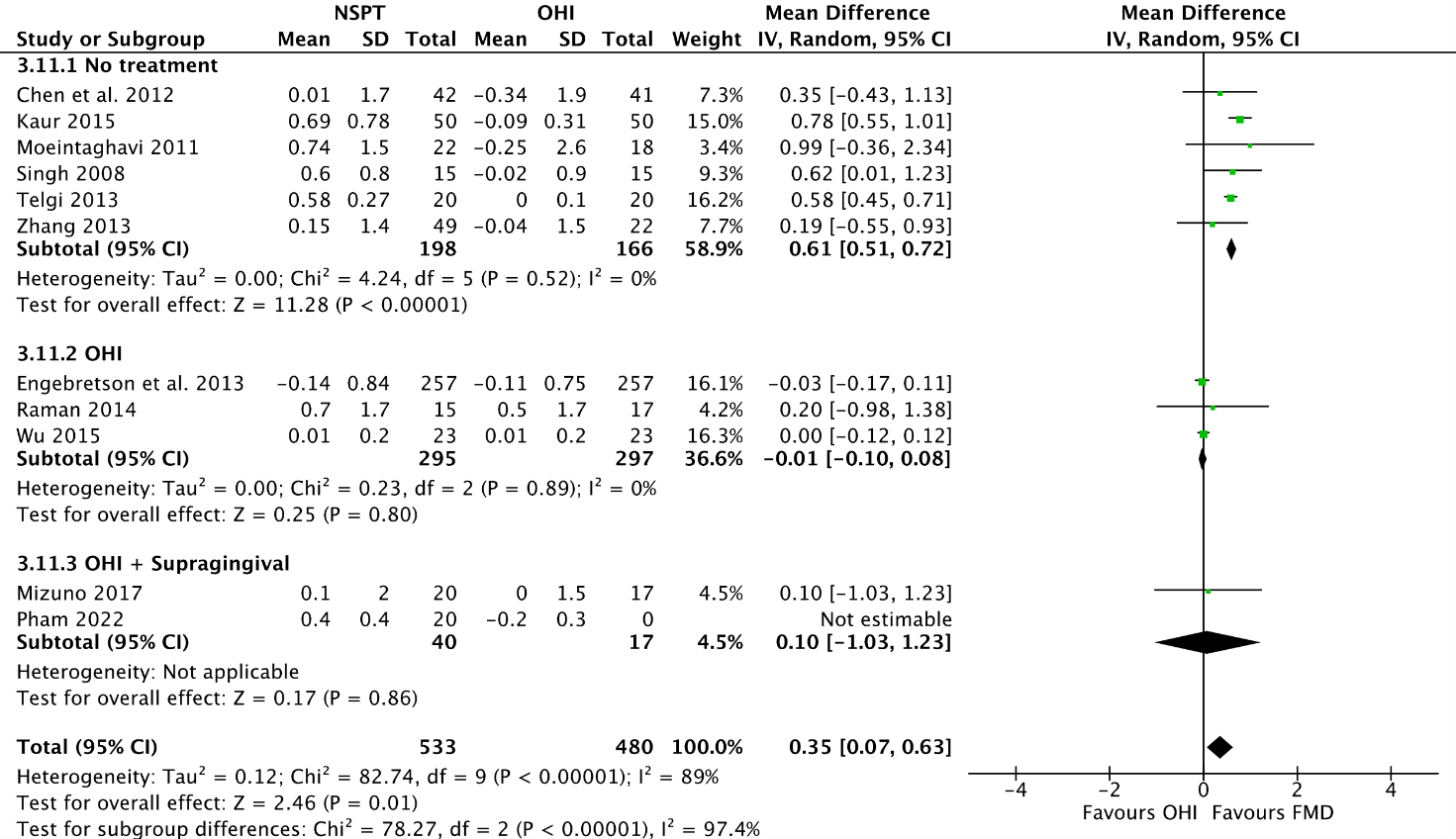


OHI / No treatment vs NSPT (HbA1c% reduction – 6 months)


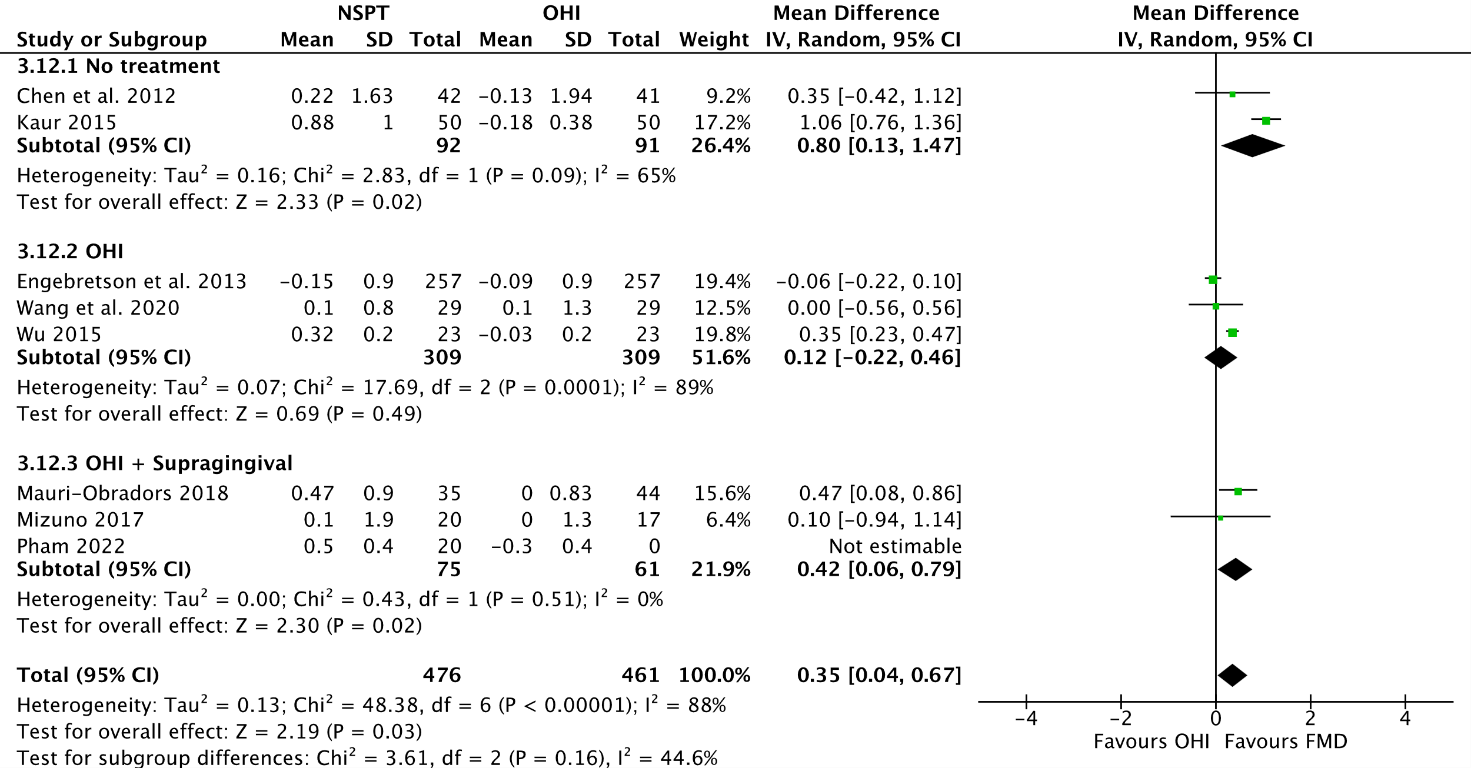


NSPT with FMD protocol vs NSPT with non-FMD protocol (PPD reduction – 3 months)


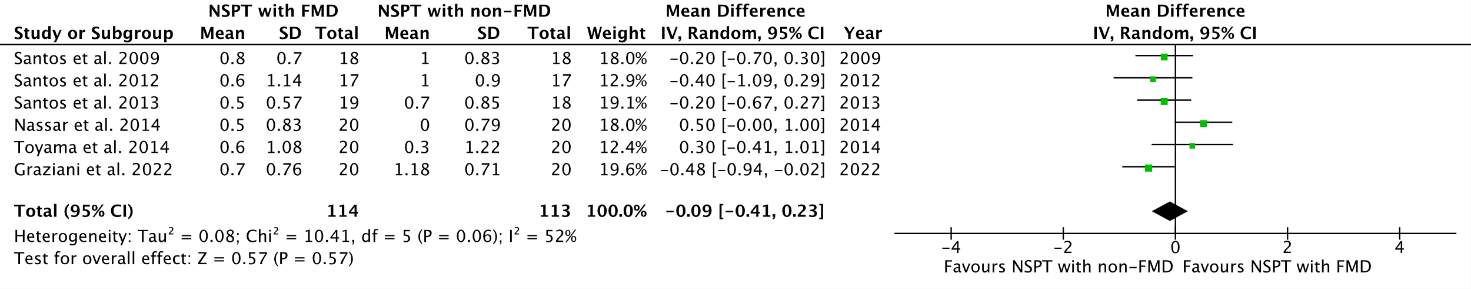


NSPT with FMD protocol vs NSPT with non-FMD protocol (PPD reduction – 6 months)

**
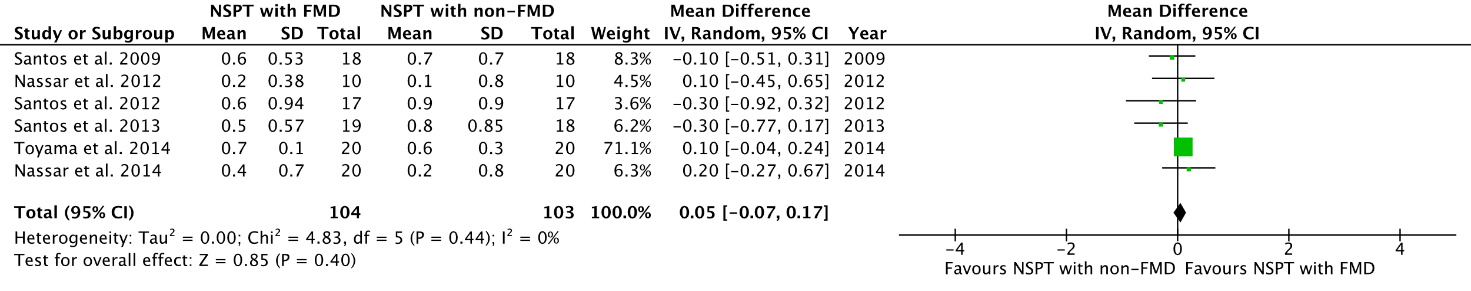
**

NSPT with FMD protocol vs NSPT with non-FMD protocol (CAL gain – 3 months)

**
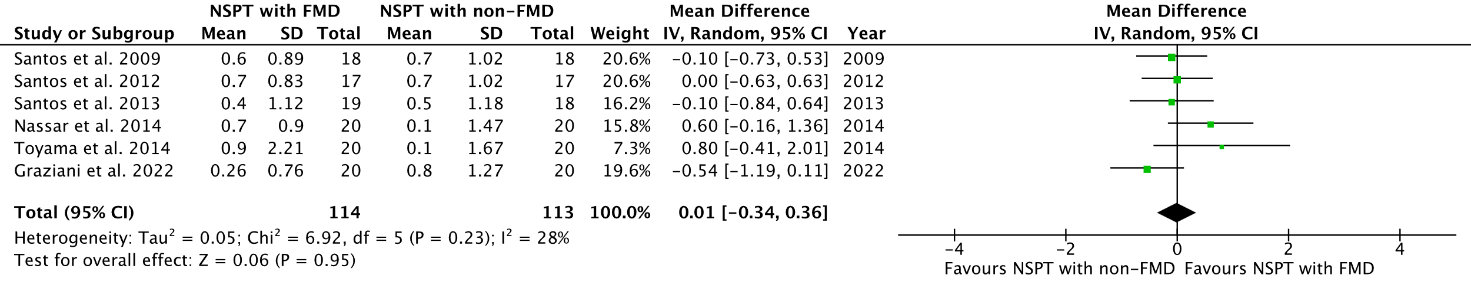
**

NSPT with FMD protocol vs NSPT with non-FMD protocol (CAL gain – 6 months)


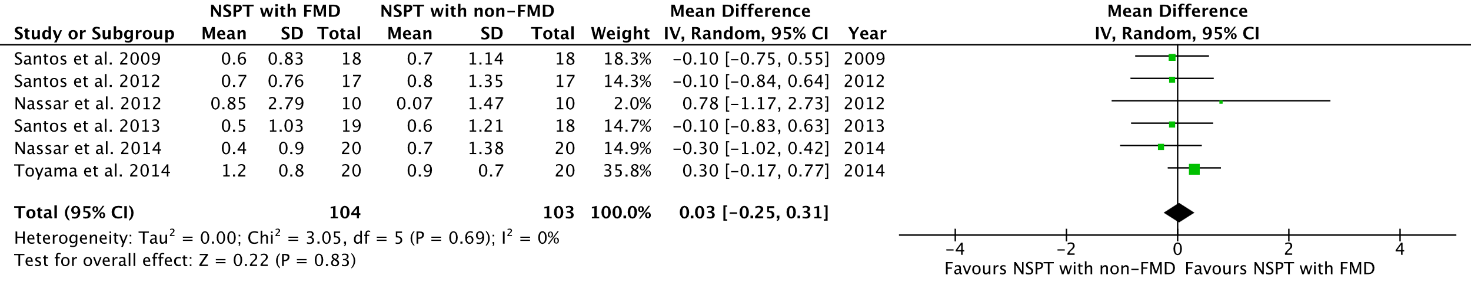


NSPT with FMD protocol vs NSPT with non-FMD protocol (BoP% reduction – 3 months)


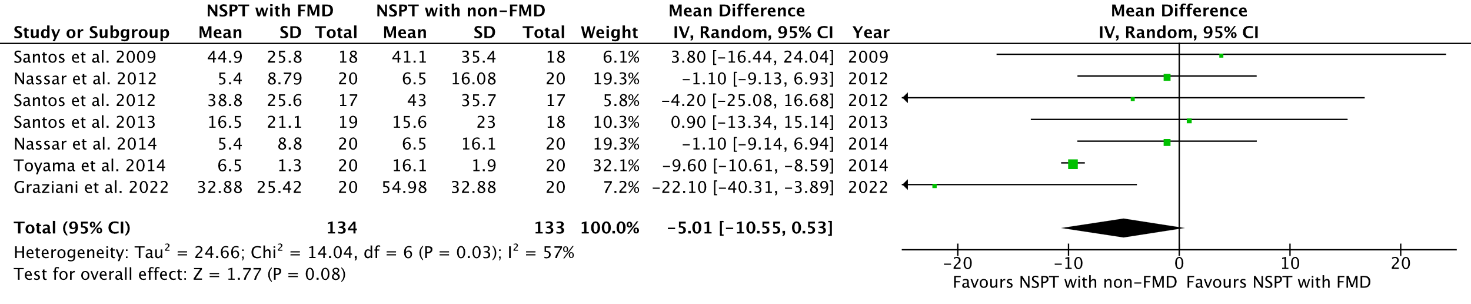


NSPT with FMD protocol vs NSPT with non-FMD protocol (BoP% reduction – 6 months)


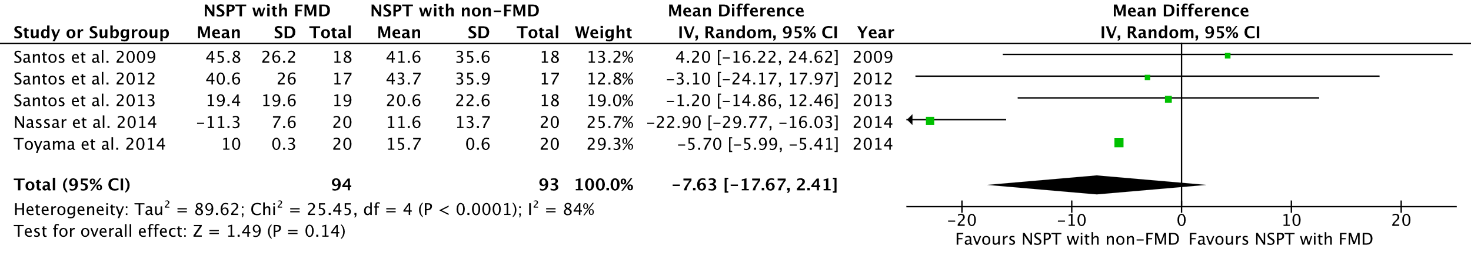


NSPT with FMD protocol vs NSPT with non-FMD protocol (HbA1c reduction – 3 months)


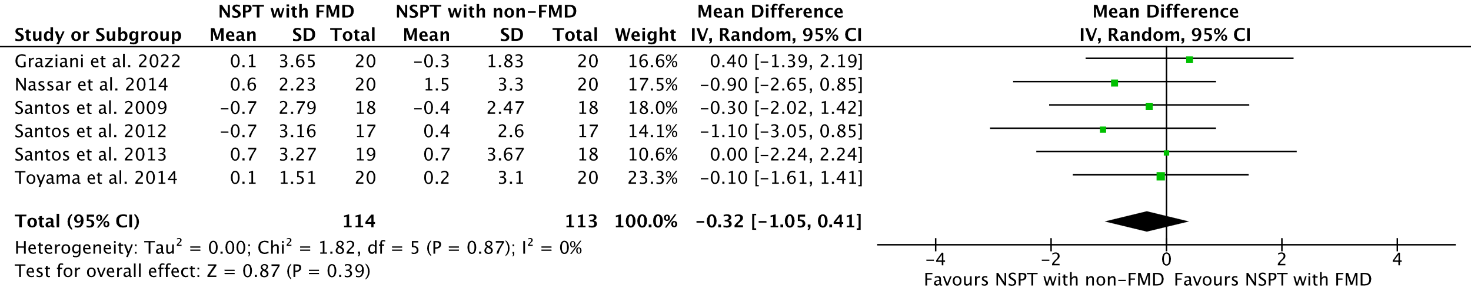


NSPT with FMD protocol vs NSPT with non-FMD protocol (HbA1c reduction – 6 months)


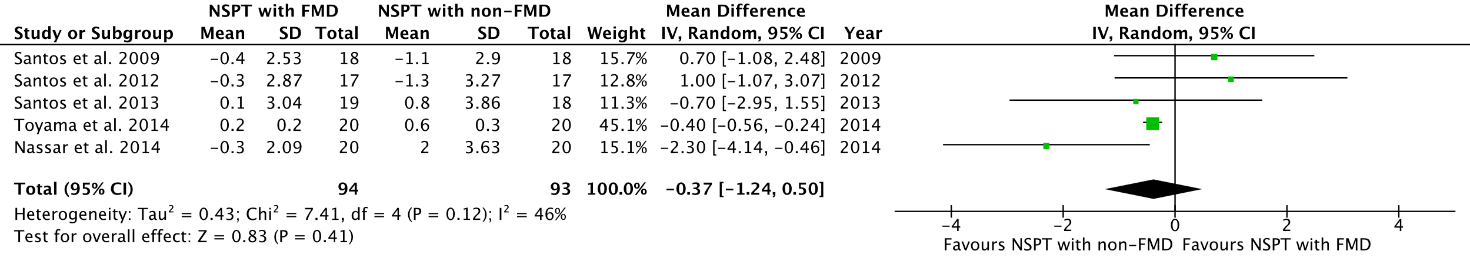


NSPT with FMD protocol vs NSPT with non-FMD protocol (%PPD ≤ 5mm reduction – 3 months)


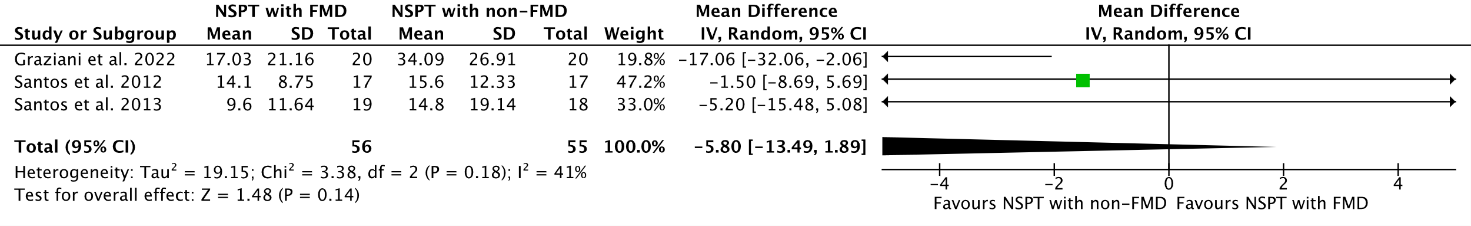


Sensitivity analysis without studies using CHX - OHI / No treatment vs NSPT (PPD reduction – 3 months)


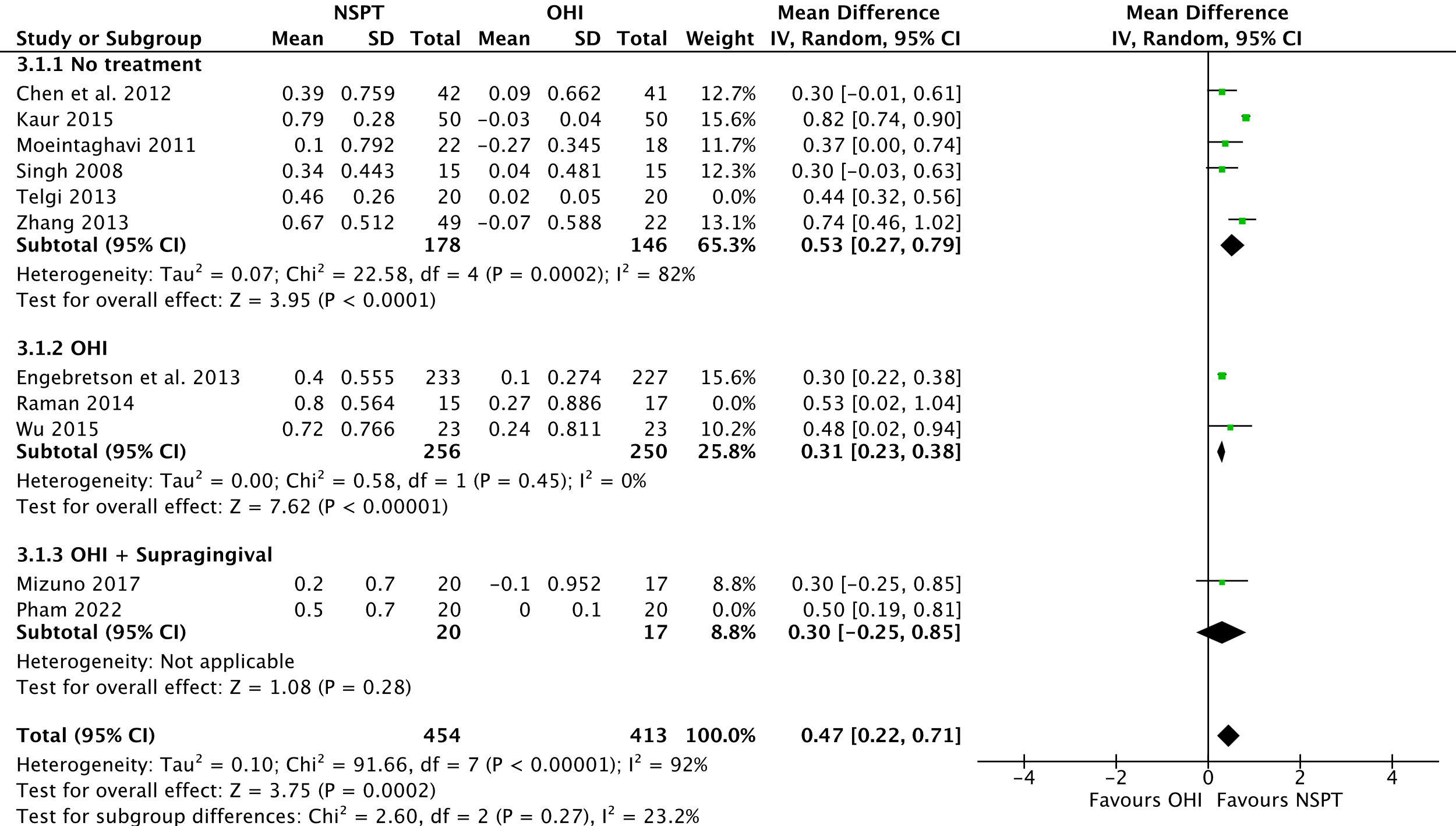


Sensitivity analysis without studies using CHX - OHI / No treatment vs NSPT (PPD reduction – 6 months)


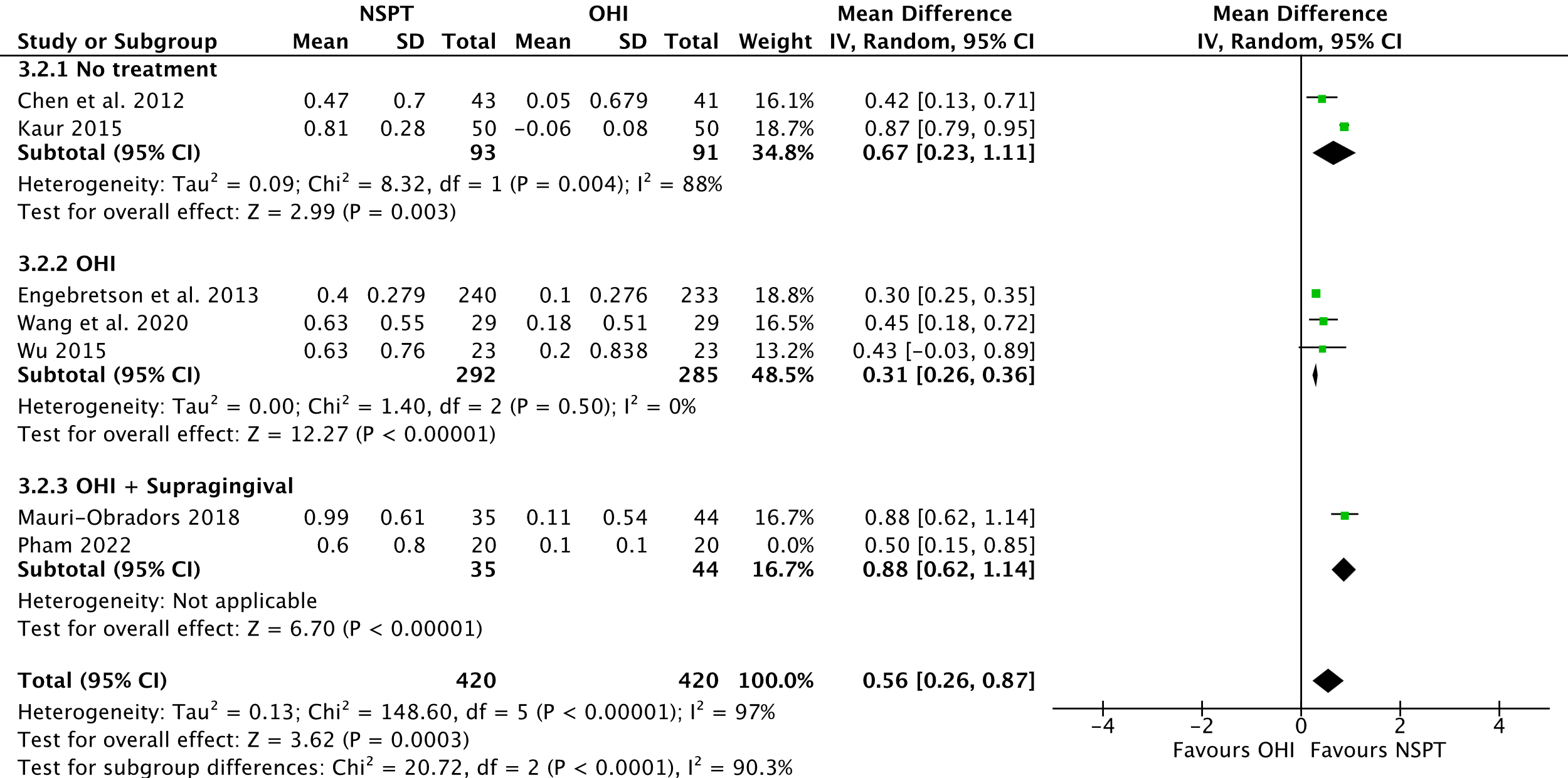


Sensitivity analysis without studies using CHX - OHI / No treatment vs NSPT (CAL reduction – 3 months)


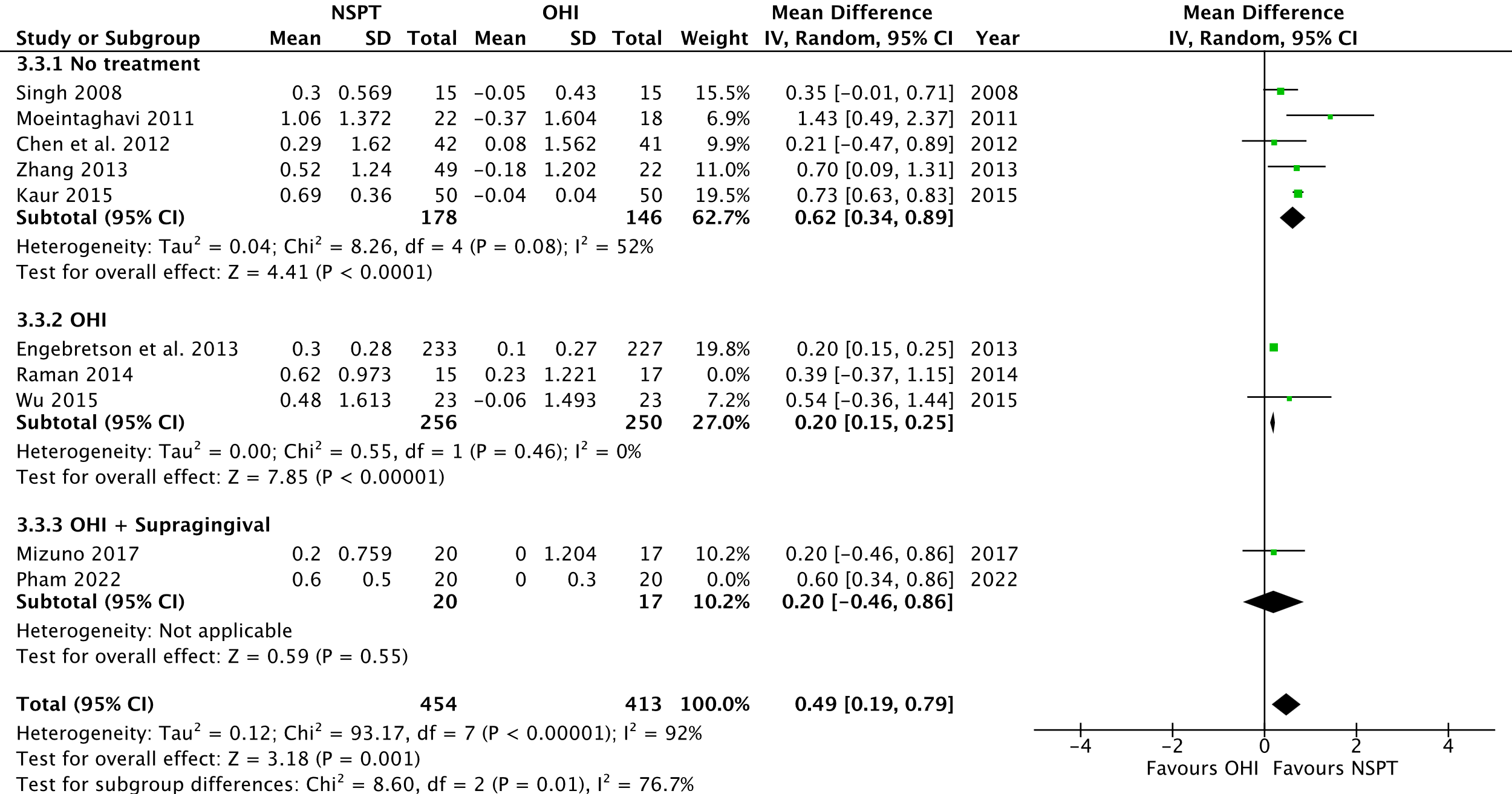


Sensitivity analysis without studies using CHX - OHI / No treatment vs NSPT (CAL reduction – 6 months)


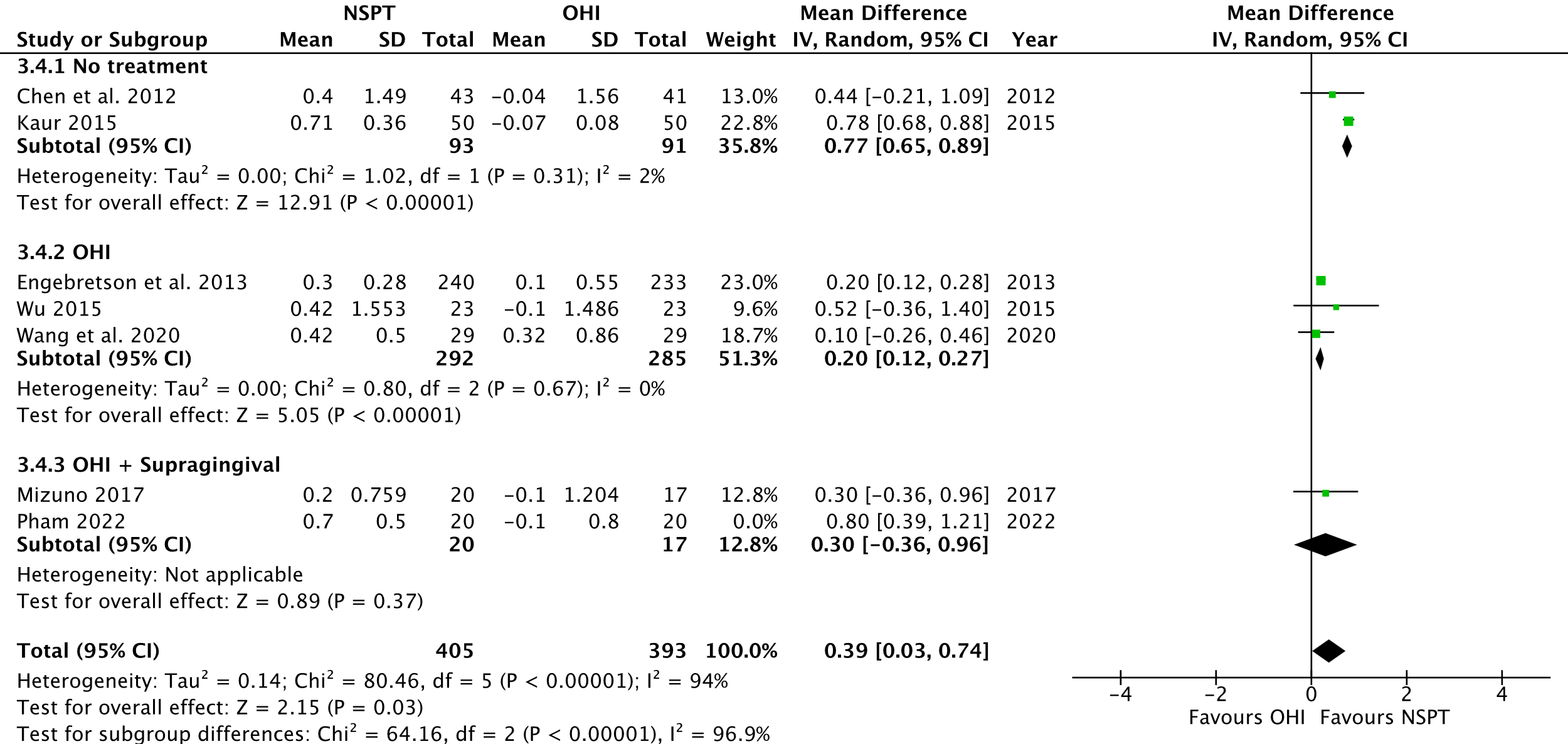


Sensitivity analysis without studies using CHX - OHI / No treatment vs NSPT (BoP% reduction – 3 months)


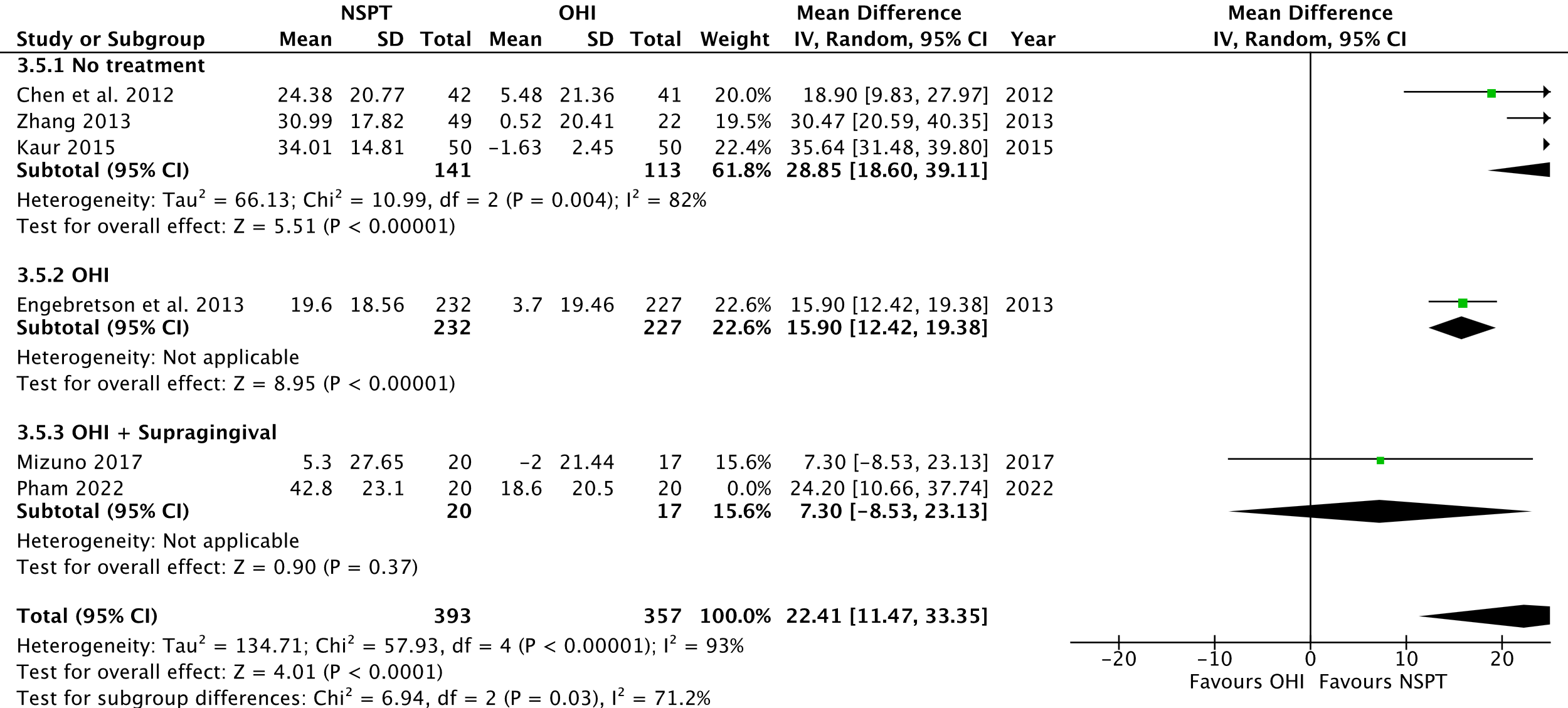


Sensitivity analysis without studies using CHX - OHI / No treatment vs NSPT (BoP% reduction – 6 months)


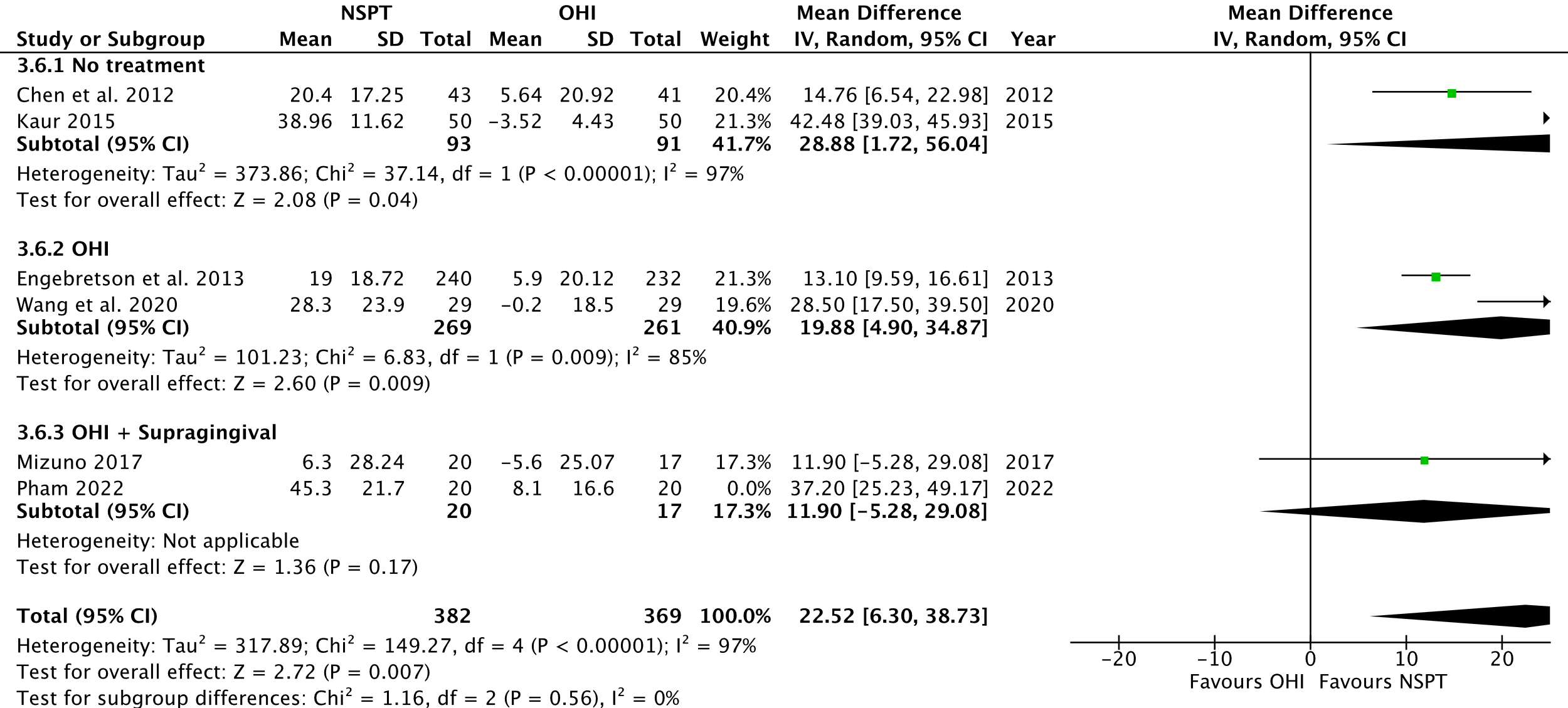


Sensitivity analysis without studies using CHX - OHI / No treatment vs NSPT (GI reduction – 3 months)


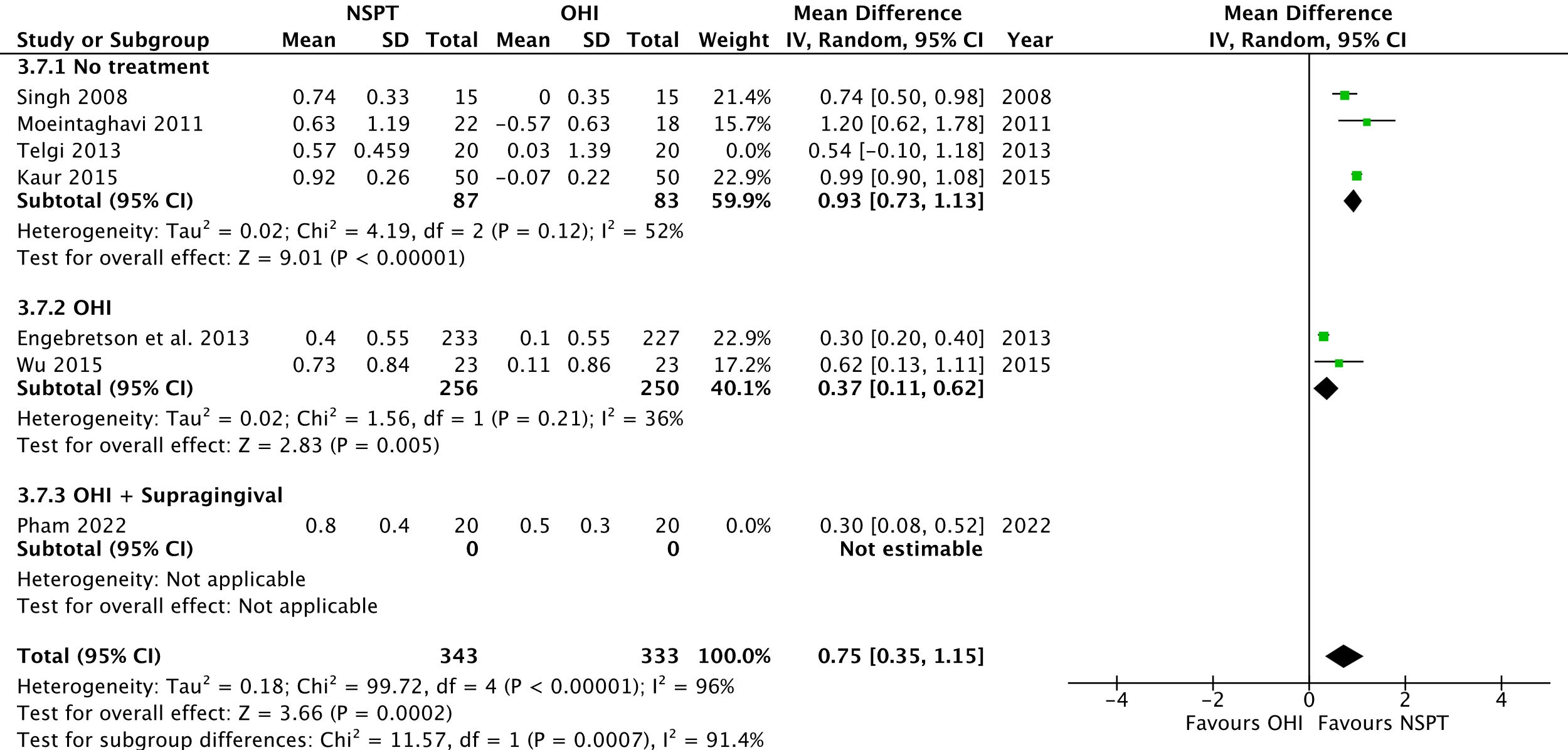


Sensitivity analysis without studies using CHX - OHI / No treatment vs NSPT (GI reduction – 6 months)


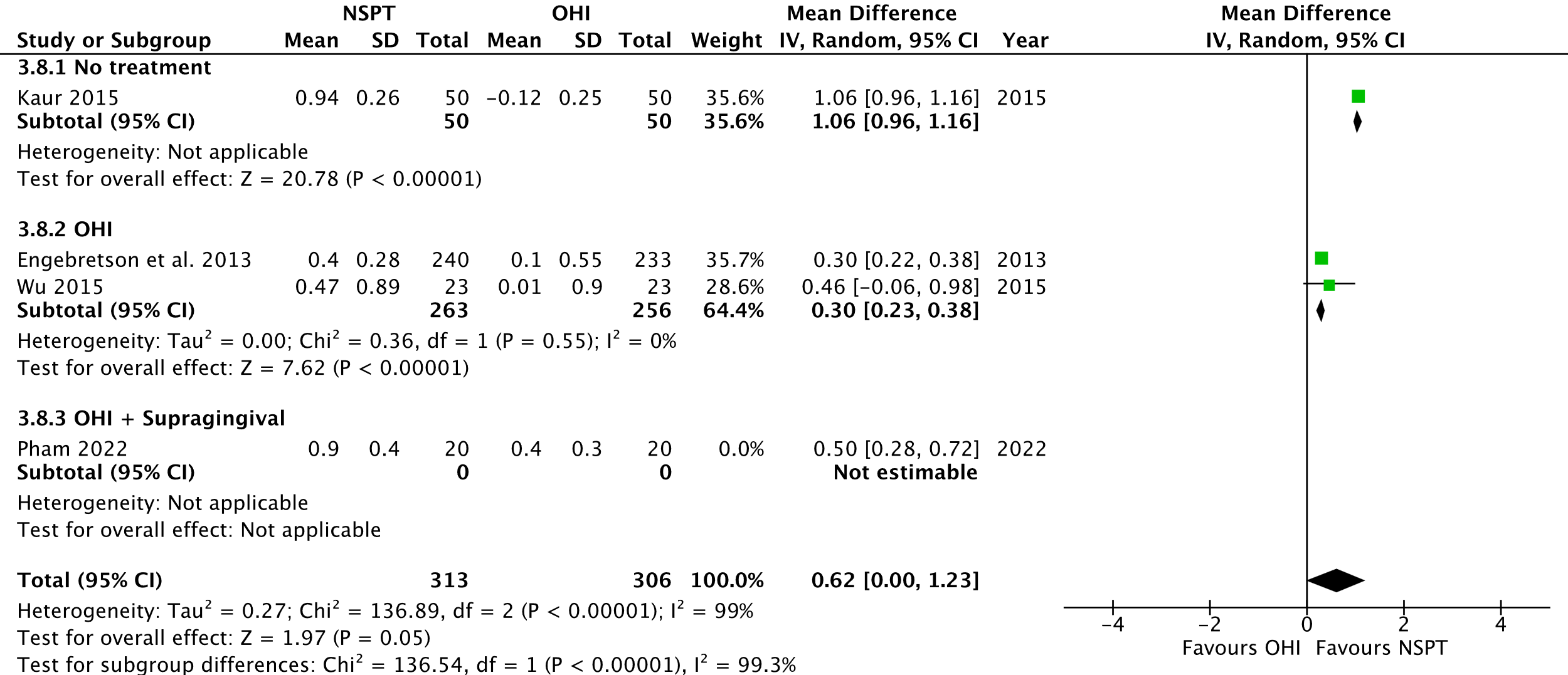


Sensitivity analysis without studies using CHX - OHI / No treatment vs NSPT (PI reduction – 3 months)


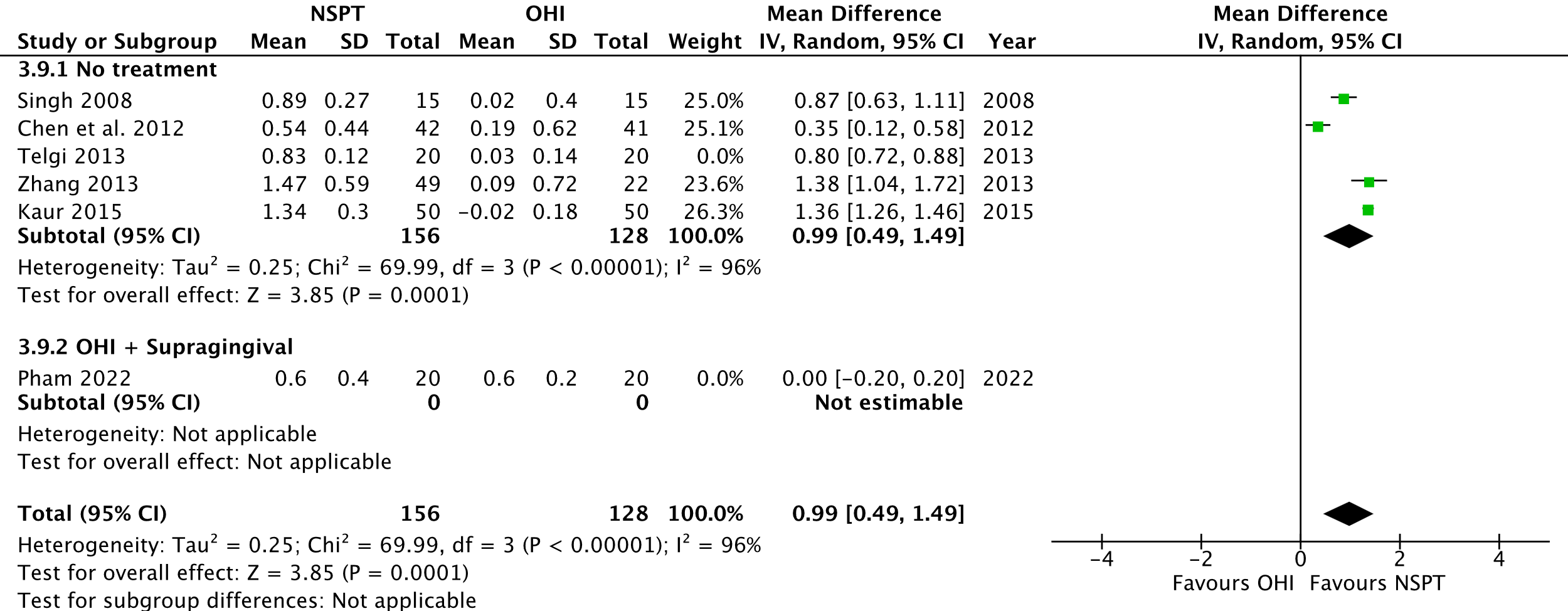


Sensitivity analysis without studies using CHX - OHI / No treatment vs NSPT (PI% reduction – 3 months)

**
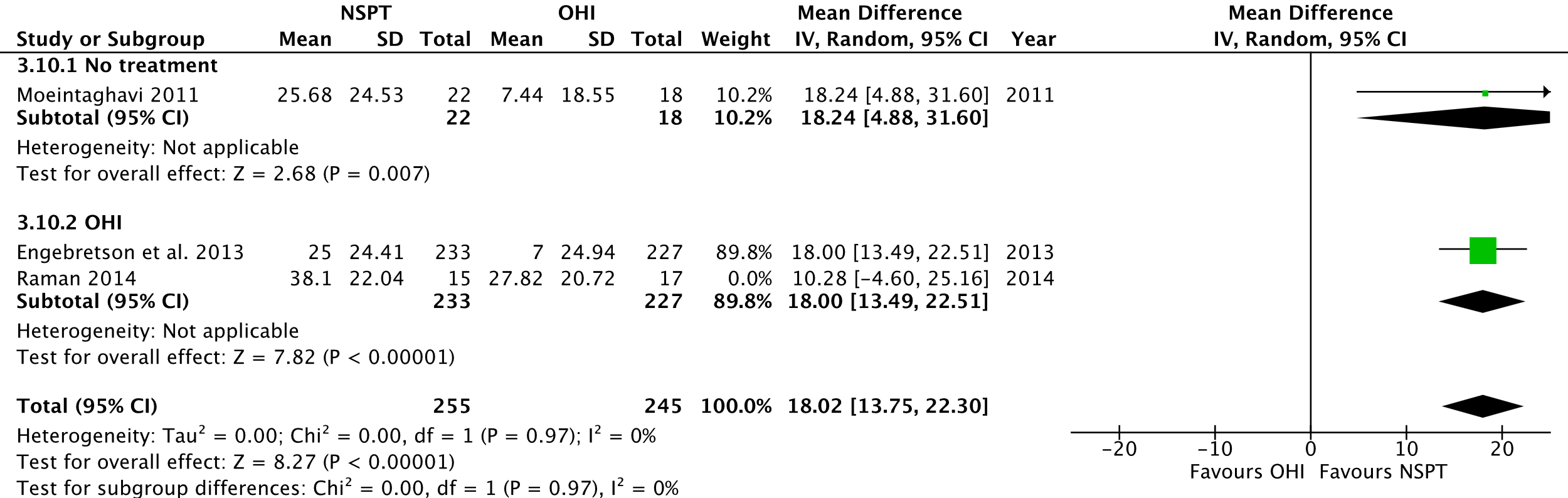
**

Sensitivity analysis without studies using CHX - OHI / No treatment vs NSPT (HbA1c reduction – 3 months)

**
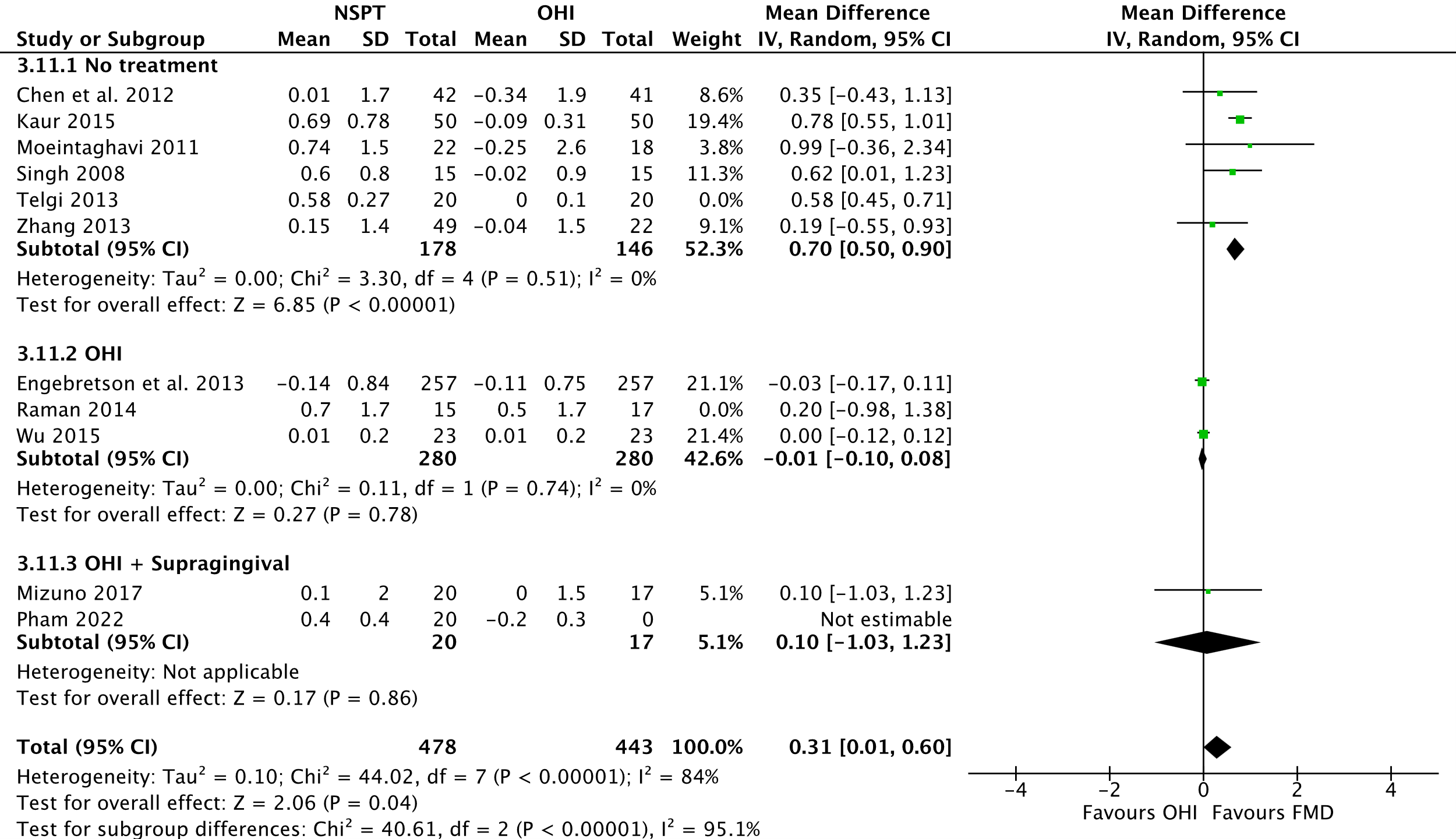
**

Sensitivity analysis without studies using CHX - OHI / No treatment vs NSPT (HbA1c reduction – 6 months)

**
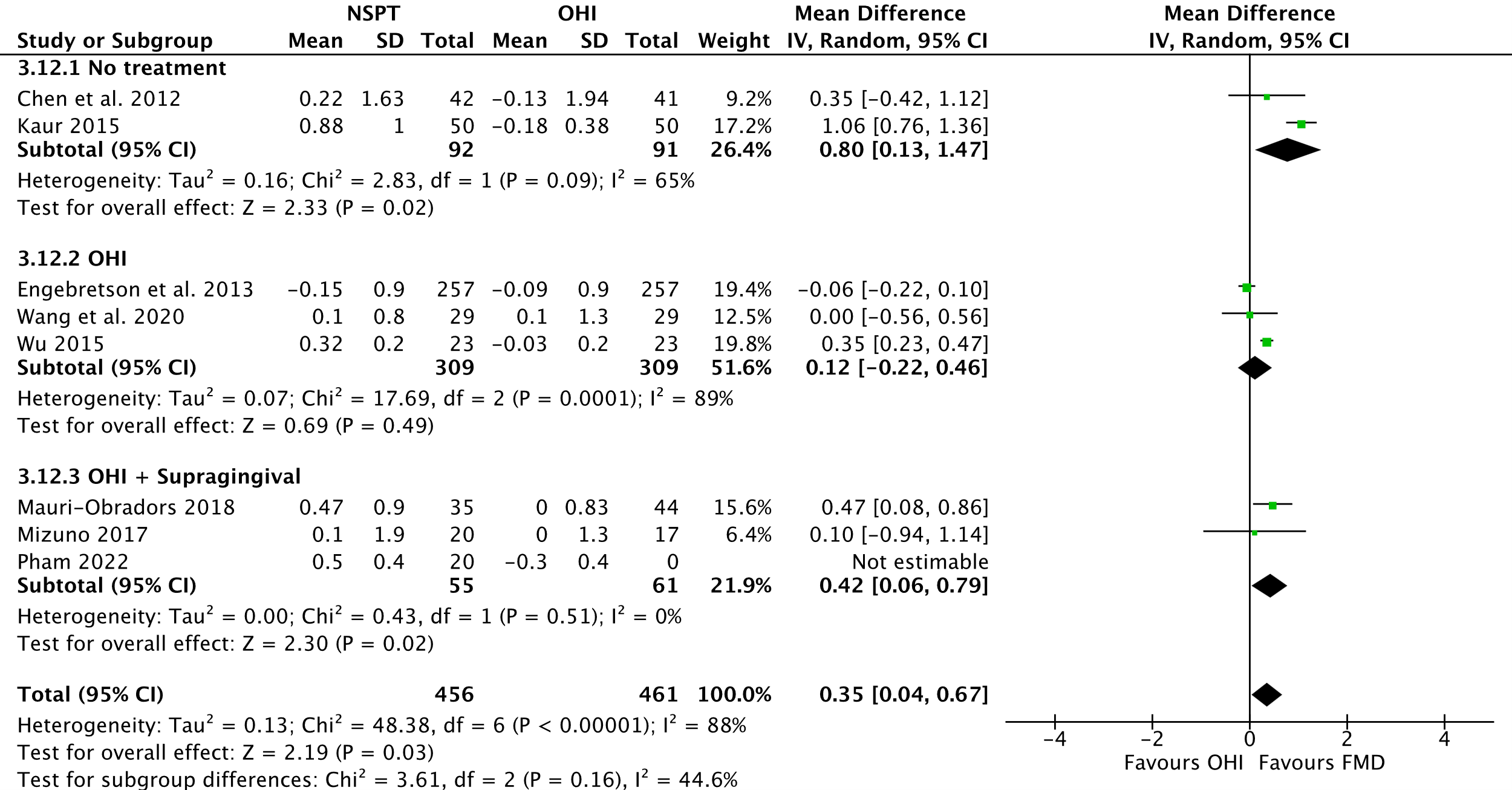
**

Sensitivity analysis without studies using CHX - NSPT with FMD protocol vs NSPT with non-FMD protocol (PPD reduction – 3 months)


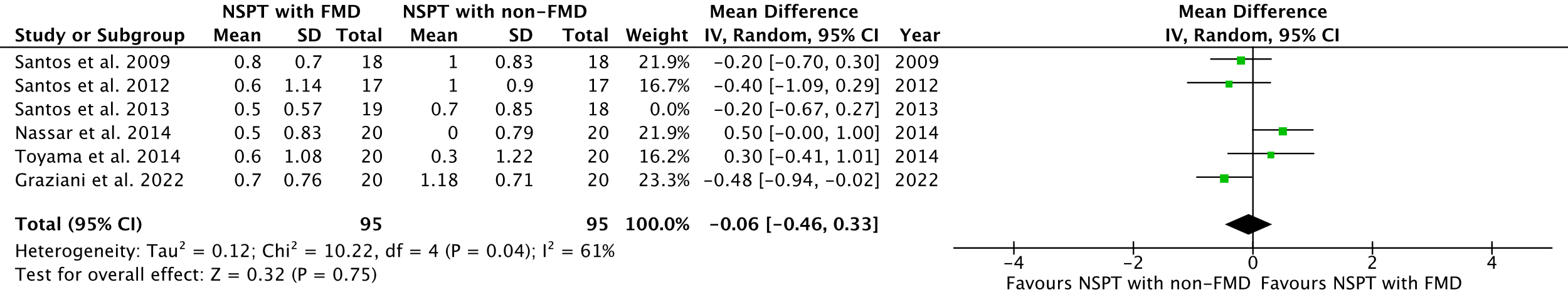


Sensitivity analysis without studies using CHX - NSPT with FMD protocol vs NSPT with non-FMD protocol (PPD reduction – 6 months)


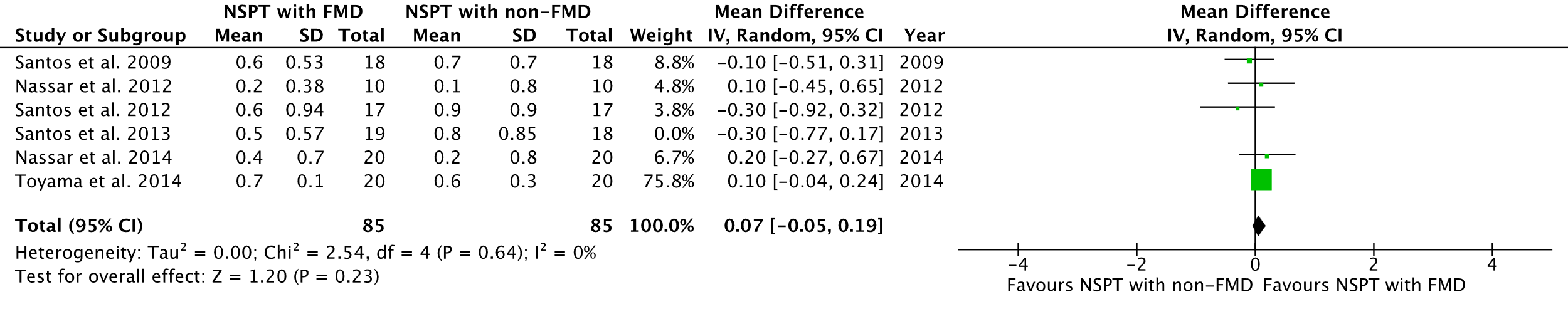


Sensitivity analysis without studies using CHX - NSPT with FMD protocol vs NSPT with non-FMD protocol (CAL reduction – 3 months)


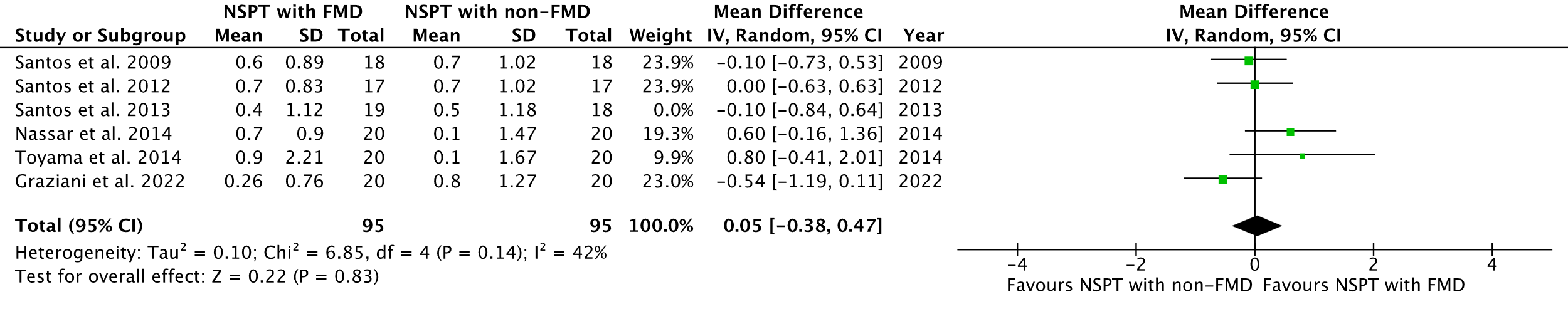


Sensitivity analysis without studies using CHX - NSPT with FMD protocol vs NSPT with non-FMD protocol (CAL reduction – 6 months)


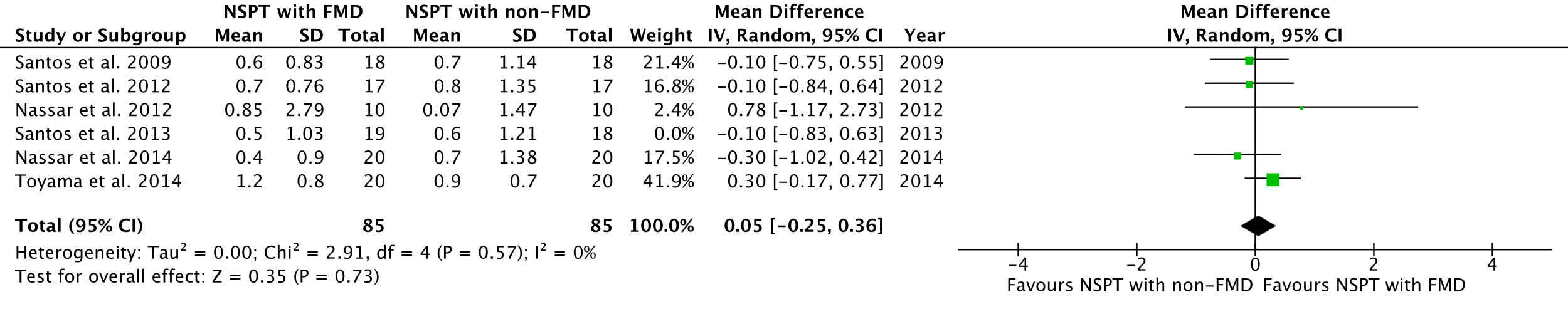


Sensitivity analysis without studies using CHX - NSPT with FMD protocol vs NSPT with non-FMD protocol (BoP% reduction – 3 months)


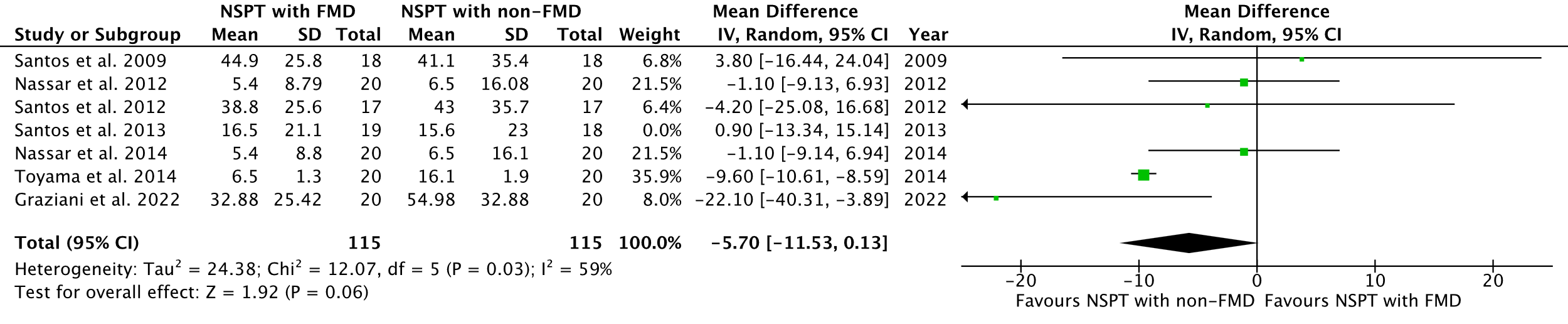


Sensitivity analysis without studies using CHX - NSPT with FMD protocol vs NSPT with non-FMD protocol (BoP% reduction – 6 months)


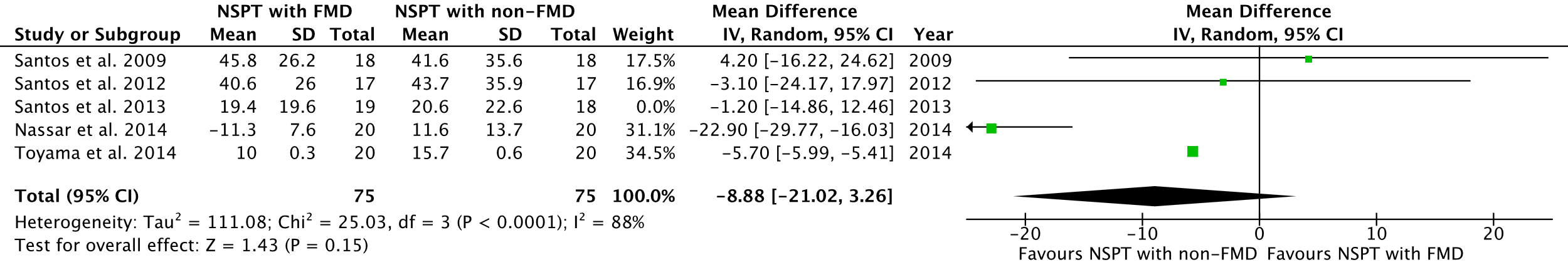


Sensitivity analysis without studies using CHX - NSPT with FMD protocol vs NSPT with non-FMD protocol (HbA1c reduction – 3 months)


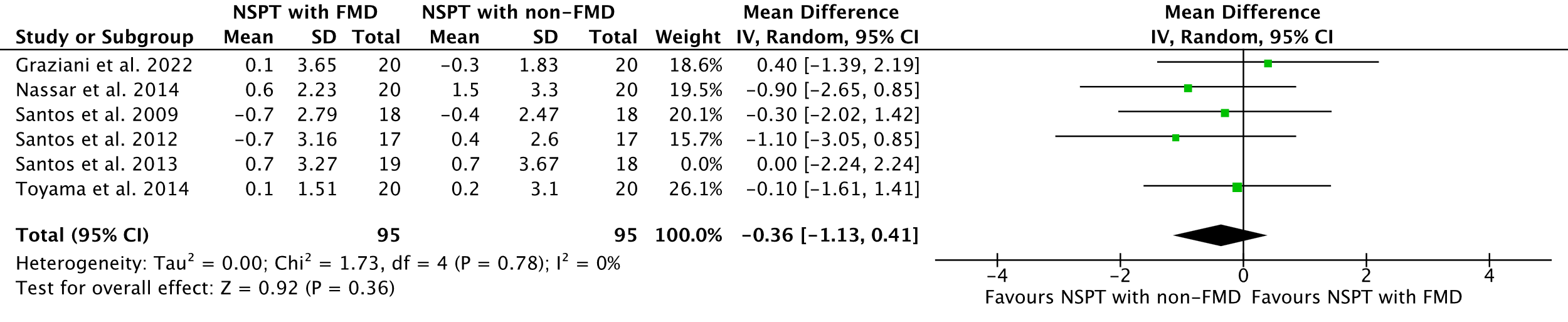


Sensitivity analysis without studies using CHX - NSPT with FMD protocol vs NSPT with non-FMD protocol (HbA1c reduction – 6 months)

**
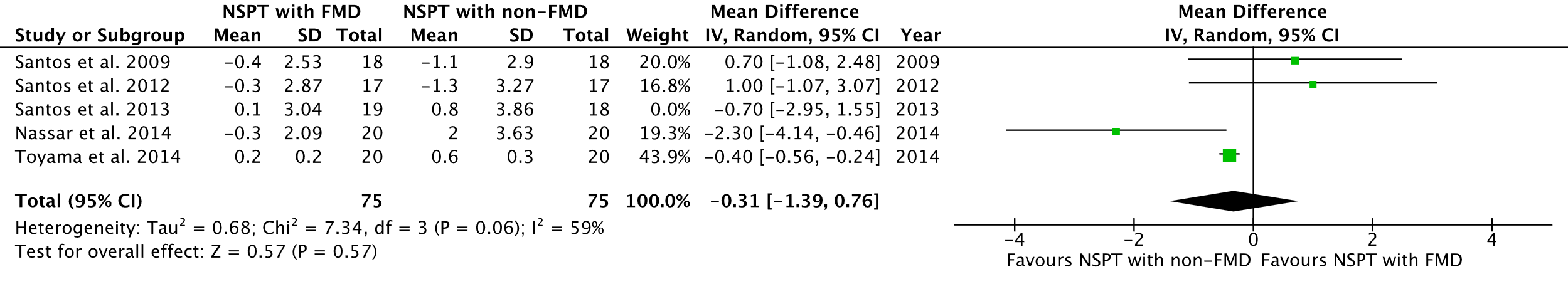
**

Sensitivity analysis without studies using CHX - NSPT with FMD protocol vs NSPT with non-FMD protocol (%PPD>=5 mm reduction – 3 months)

**
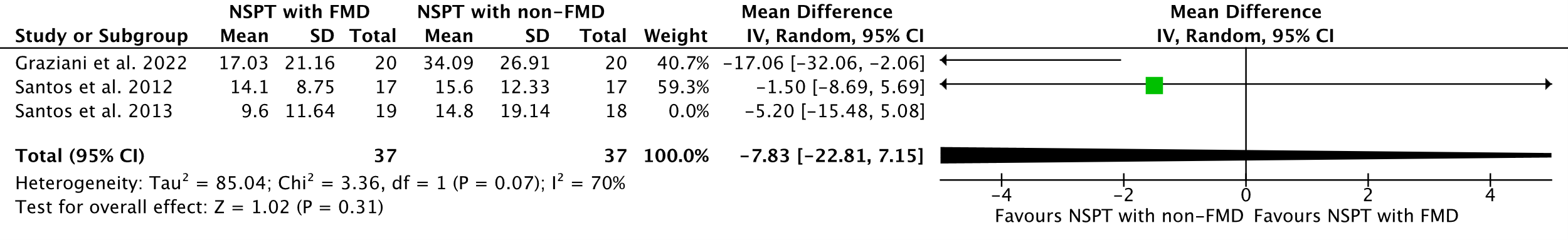
**

**Appendix S5**

**Results of the sensitivity analysis excluding the studies using CHX**

|  |  | **3 months** | | | **6 months** | | |
| --- | --- | --- | --- | --- | --- | --- | --- |
|  |  | *Mean [95% CI] (n° of studies)* | *P* | *I^2^* | *Mean [95% CI] (n° of studies)* | *P* | *I^2^* |
| **OHI / No treatment vs NSPT** | *PPD reduction* | 0.47 [0.22, 0.71] (8)  No treatment: 0.53 [0.27, 0.79] (5)  OHI: 0.31 [0.23, 0.38] (2)  OHI + Supragingival: 0.30 [-0.25, 0.85] (1) | **< 0.001**  **No treatment: < 0.001**  OHI: 0.45  OHI + Supragingival: = 0.28 | **92%**  **No treatment: 82%**  OHI: 0%  OHI + Supragingival: N/A | 0.56 [0.26, 0.87] (6)  No treatment: 0.67 [0.23, 1.11] (2)  OHI: 0.31 [0.26, 0.36] (3)  OHI + Supragingival: 0.88 [0.62, 1.14] (1) | **< 0.001**  **No treatment: 0.004**  OHI: 0.50  **OHI + Supragingival: < 0.001** | **97%**  **No treatment: 88%**  OHI: 0%  **OHI + Supragingival: N/A** |
|  | *CAL gain* | 0.49 [0.19, 0.79] (8)  No treatment: 0.62 [0.34, 0.89] (5)  OHI: 0.20 [0.15, 0.25] (2)  OHI + Supragingival: 0.20 [-0.46, 0.86] (1) | **< 0.001**  N**o treatment: < 0.001**  **OHI: < 0.001**  OHI + Supragingival: = 0.55 | **92%**  **No treatment: 52%**  **OHI: 0%**  OHI + Supragingival: N/A | 0.39 [0.03, 0.74] (6)  No treatment: 0.77 [0.65, 0.89] (2)  OHI: 0.20 [0.12, 0.27] (3)  OHI + Supragingival: 0.30 [-0.36, 0.96] (1) | **< 0.001**  **No treatment: < 0.001**  **OHI: < 0.001**  OHI + Supragingival: = 0.37 | **94%**  **No treatment: 2%**  **OHI: 0%**  OHI + Supragingival: N/A |
|  | *BoP% reduction* | 22.41 [11.47, 33.35] (5)  No treatment: 28.85 [18.60, 39.11] (3)  OHI: 15.90 [12.42, 19.38] (1)  OHI + Supragingival: 7.30 [-8.53, 23.13] (1) | **< 0.001**  **No treatment: 0.004**  OHI: <0.001  OHI + Supragingival: 0.37 | **93%**  **No treatment: 82%**  OHI: N/A  OHI + Supragingival: N/A | 22.52 [6.30, 38.73] (5)  No treatment: 28.88 [1.72, 56.04] (2)  OHI: 19.88 [4.90, 34.87] (2)  OHI + Supragingival: 11.90 [-5.28, 29.08] (1) | **< 0.001**  **No treatment: 0.04**  **OHI: 0.009**  OHI + Supragingival: = 0.17 | **97%**  **No treatment: 97%**  **OHI: 85%**  OHI + Supragingival: N/A |
|  | *GI reduction* | 0.75 [0.35, 1.15] (5)  No treatment: 0.93 [0.73, 1.13] (3)  OHI: 0.37 [0.11, 0.62] (2) | **< 0.001**  No treatment: 0.12  **OHI: 0.005** | **96%**  No treatment: 52%  **OHI: 36%** | 0.62 [0.00, 1.23] (3)  No treatment: 1.06 [0.96, 1.16] (1)  OHI: 0.30 [0.23, 0.38] (2) | **< 0.001**  **No treatment: < 0.001**  **OHI: < 0.001** | **99%**  **No treatment: N/A**  **OHI: 0%** |
|  | *PI reduction* | 0.99 [0.49, 1.49] (4)  No treatment: 0.99 [0.49, 1.49] (4) | **< 0.001**  **No treatment: < 0.001** | **96%**  **No treatment: 96%** | - | - | - |
|  | *PI% reduction* | 18.02 [13.75, 22.30] (2)  No treatment: 18.24 [4.88, 31.60] (1)  OHI: 18.00 [13.49, 22.51] (1) | **< 0.001**  **No treatment: 0.007**  **OHI: < 0.001** | **0%**  **No treatment: N/A**  **OHI: N/A** | - | - | - |
|  | *HbA1c reduction* | 0.31 [0.01, 0.60] (8)  No treatment: 0.70 [0.50, 0.90] (5)  OHI: -0.01 [-0.10, 0.08] (2)  OHI + Supragingival: 0.10 [-1.03, 1.23] (1) | **0.04**  **No treatment: < 0.001**  OHI: 0.78  OHI + Supragingival: 0.86 | **84%**  **No treatment: 0%**  OHI: 0%  OHI + Supragingival: N/A | 0.35 [0.04, 0.67] (8)  No treatment: 0.80 [0.13, 1.47] (2)  OHI: 0.12 [-0.22, 0.46] (3)  OHI + Supragingival: 0.42 [0.06, 0.79] (2) | **0.03**  **No treatment: 0.02**  **OHI: 0.49**  **OHI + Supragingival: 0.02** | **88%**  **No treatment: 65%**  **OHI: 89%**  **OHI + Supragingival: 0%** |
| **NSPT with FMD protocol vs NSPT with non-FMD protocol** | *PPD reduction* | -0.06 [-0.46, 0.33] (5) | 0.75 | 61% | 0.07 [-0.05, 0.19] (5) | 0.23 | 0% |
|  | *CAL gain* | 0.05 [-0.38, 0.47] (5) | 0.83 | 42% | 0.05 [-0.25, 0.36] (5) | 0.73 | 0% |
|  | *BoP% reduction* | -5.70 [-11.53, 0.13] (6) | 0.06 | 59% | -8.88 [-21.02, 3.26] (4) | 0.15 | 88% |
|  | *HbA1c reduction* | -0.36 [-1.13, 0.41] (5) | 0.36 | 0% | -0.31 [-1.39, 0.76] (5) | 0.57 | 59% |
|  | *% sites with PPD ≥ 5 mm* | -7.83 [-22.81, 7.15] (2) | 0.31 | 70% | - | - | - |
